# Large size (>100‐μm) microplastics are not biomagnifying in coastal marine food webs of British Columbia, Canada

**DOI:** 10.1002/eap.2654

**Published:** 2022-07-04

**Authors:** Garth A. Covernton, Kieran D. Cox, Wendy L. Fleming, Brittany M. Buirs, Hailey L. Davies, Francis Juanes, Sarah E. Dudas, John F. Dower

**Affiliations:** ^1^ Department of Biology University of Victoria Victoria British Columbia Canada; ^2^ Present address: Department of Ecology and Evolutionary Biology University of Toronto Toronto Ontario Canada; ^3^ Hakai Institute Calvert Island British Columbia Canada; ^4^ Fisheries and Oceans Canada Pacific Biological Station Nanaimo British Columbia Canada; ^5^ School of Earth and Ocean Sciences University of Victoria Victoria British Columbia Canada

**Keywords:** Bayesian, bioaccumulation, digestive tracts, ecotoxicology, fish, food webs, ingestion, invertebrates, livers, machine learning, trophic

## Abstract

Microplastics (MPs) contamination in marine environments is of increasing concern, as plastic particles are globally ubiquitous across ecosystems. A large variety of aquatic taxa ingest MPs, but the extent to which animals accumulate and transfer MPs through food webs is largely unknown. In this study, we quantified MP uptake in bivalves, crabs, echinoderms, and fish feeding at different trophic levels at three sites on southern Vancouver Island. We paired stable‐isotope food web analysis with MP concentrations in digestive tracts across all trophic levels and in fish livers. We then used Bayesian generalized linear mixed models to explore whether bioaccumulation and biomagnification were occurring. Our results showed that MPs (100–5000 μm along their longest dimension) are not biomagnifying in marine coastal food webs, with no correlation between the digestive tract or fish liver MP concentrations and trophic position of the various species. Ecological traits did, however, affect microplastic accumulation in digestive tracts, with suspension feeder and smaller‐bodied planktivorous fish ingesting more MPs by body weight. Trophic transfer occurred between prey and predator for rockfish, but higher concentrations in full stomachs compared with empty ones suggested rapid excretion of ingested MPs. Collectively, our findings suggested the movement of MP through marine food webs is facilitated by species‐specific mechanisms, with contamination susceptibility a function of species biology, not trophic position. Furthermore, the statistical methods we employ, including machine learning for classifying unknown particles and a probabilistic way to account for background contamination, are universally applicable to the study of microplastics. Our findings advance understanding of how MPs enter and move through aquatic food webs, suggesting that lower‐trophic‐level animals are more at risk of ingesting >100‐μm MPs, relative to higher‐trophic‐level animals. Our work also highlights the need to advance the study of <100‐μm MPs, which are still poorly understood and may need to be considered separately in ecological risk assessments.

## INTRODUCTION

Microplastics (MPs) are complex and uniquitous contaminants that occur in every environmental compartment (Rochman et al., 2019; Zhang, Jiuqi Wang, et al., 2019). Although numerous studies have documented the presence of MPs in the environment and various animals, our understanding of the risks that MPs pose to aquatic and terrestrial communities remains limited. From a toxicological standpoint, risk is proportionate to the product of hazard and exposure, where the former represents the potential for harm and the latter depends on dose and duration. Risk can also manifest on different time and biological scales of organization, including immediate effects on organisms and populations (e.g., changes in growth, reproduction, or mortality), or as chronic shifts in behavior or metabolism that alter energy budgets, species interactions, or intergenerational effects (Fleeger et al., 2003; Muller et al., 2010; Skinner et al., 2011). Thus, as part of any ecosystem‐level risk assessment for MPs, the routes and extent of exposure for different organisms (e.g., relative exposure from diet vs. respiration and the contribution from different food sources) are important aspects to consider. Because ingestion represents the main pathway by which MPs enter the bodies of animals (Pinheiro et al., 2020), understanding the feeding habits and trophic positions of animals is vital to quantifying their exposure to MPs.

Within the field of ecotoxicology, bioaccumulation and biomagnification are two of the most important metrics for determining ecological risk, as they are key predictors of exposure. Bioaccumulation commonly refers to the relative rates that a contaminant enters and exits the body of an animal (Gobas et al., 1988). If excretion or chemical decomposition occurs faster than entry rates, no bioaccumulation occurs. For example, many bioaccumulating chemicals are lipophilic and are thus sequestered in the body and prevented from being excreted, although this process does not necessarily occur equally throughout the body and can be organ‐specific (Piscopo et al., 2016). Biomagnification occurs when a contaminant accumulates in the body of an animal and is then transferred to its predator. This process results in increasing levels of exposure for individuals with increasing trophic position (Kelly et al., 2007).

There are several commonly used metrics for quantifying the processes of bioaccumulation and biomagnification. The bioaccumulation factor can be measured in the field as the steady‐state ratio of the concentration of a contaminant in an animal (commonly grams per kilogram [g kg^−1^]) relative to that in the surrounding water (commonly grams per liter [g L^−1^]), representing the accumulation of a contaminant from all potential sources in the environment, including diet (Arnot & Gobas, 2006; Borgå et al., 2004; Gobas et al., 2009). MPs are often difficult to separate completely from all other materials and difficult to weigh, so concentrations are commonly expressed in terms of the number of particles rather than their mass. However, this may overlook the fragmentation of ingested particles within animal bodies. The trophic magnification factor refers to the coefficient, or slope, of a regression between the log concentration of a contaminant in an organism and its trophic position (Borgå et al., 2011; Gobas et al., 2009). Another commonly used metric is the biomagnification factor, defined as the steady‐state ratio of the concentration of a contaminant in an organism relative to the concentration in its diet (Conder et al., 2012; Gobas et al., 2009). In this study, we focus on the bioaccumulation and trophic magnification factors because we did not conduct a thorough inventory of the diet of the studied organisms. Quantifying and comparing these metrics within aquatic food webs can help determine the role of individual and trophic dynamics of animals ingesting MPs and the relative degree to which animals with different feeding habits might be at risk.

Many studies have reported on the entry of MPs into food webs and their trophic transfer, including to upper‐trophic‐level predators, but magnification has not been confirmed (Carbery et al., 2018; Nelms et al., 2018; Santana et al., 2017; Welden et al., 2018). Most studies have investigated trophic transfer using laboratory experiments, although some modeling and field work conducted in Canada, namely an Ontario lake and in the northeast Pacific Ocean, suggest that trophic magnification is not occurring (Alava, 2020; McIlwraith et al., 2021). Some recent studies have suggested that factors other than trophic level may be more important for determining the ingestion and accumulation rates of MPs in marine animals (Covernton et al., 2021; Gouin, 2020; Miller et al., 2020; Walkinshaw et al., 2020). These factors include MP elimination rates for organisms of different species and life‐history stages, differences in feeding habits, degree of contamination of the surrounding environment, and the probability of encountering a MP within a heterogeneous local environment (Güven et al., 2017; Santana et al., 2017; Setälä et al., 2016). For example, different fish feeding on sedentary epibenthic versus mobile pelagic secondary consumer invertebrates would technically be feeding at the same trophic level but using different feeding habits in different environments. Furthermore, most studies focus only on the digestive tracts of animals and use methods limited to larger MPs (>~100 μm). Smaller MPs may be able to translocate from digestive tracts into other animal tissues—likely limited to MPs <130 μm and mainly those under 10 μm in size (De Sales‐Ribeiro et al., 2020; Kim et al., 2020; Zeytin et al., 2020). There is some evidence, however, that even larger MPs have the potential to translocate to the livers of marine fish, including 214‐μm particles (mean size) in gilt‐head seabream (*Sparus aurata*) and 124‐ to 438‐μm particles in European anchovies (*Engraulis encrasicolus*) (Collard et al., 2017; Jovanović et al., 2018). Owing to the methodology used in our study—manual particle selection and spectroscopy, as opposed to automated scanning or thermoanalytical methods—this investigation is limited to particles >100 μm in size.

Despite the large number of investigations into the presence of MPs in individual species, few studies have documented the concentrations within multiple species of varying life history and feeding strategies within the same food web. Although a handful of MP studies have estimated trophic levels for distinct species, few have used quantitative food‐web ecology methods to determine individual animals' trophic positions or to characterize their role within a food web. These methods, including stable‐isotope analysis, are commonly employed by ecotoxicologists to study the accumulation and biomagnification of other organic pollutants and heavy metals (e.g., polychlorinated biphenyls, mercury) in food webs. Bioaccumulation and trophic‐magnification factors can be calculated by pairing food‐web analysis, typically using the stable‐isotope tracers ^13^C and ^15^N (Layman et al., 2012), with contaminant analysis. In the present study, we apply these methods to marine food webs in coastal British Columbia (BC), Canada, to understand the trophic dynamics of MPs in the digestive tracts of animals and the livers of fish feeding across multiple trophic levels.

We also seek to address several shortcomings in the MP field, including blank correction, incomplete spectroscopic identification of all particles, and approaches to dealing with heterogeneous, nonnormal data. Contamination from laboratory and field environments, especially by fibers, is a rampant issue in MP research (Fries et al., 2013; Woodall et al., 2015). Studies commonly deal with this issue by performing blank subtraction; however, this is a deterministic process that does not allow for uncertainty (Brander et al., 2020). Furthermore, MP data, including blank data, often take the form of positive integers with many zeros, making analysis with linear models under assumptions of normality and homogeneity of residual variance unreliable. These data are often also collected from different animals or environmental matrices that cause further heterogeneity in residual variance when simplistic models are employed. Here, we address the issues of blank correction and nonnormal, heterogeneous data using hierarchical Bayesian generalized linear mixed‐effects models that allow for uncertainty across the multiple layers of MP data, from background contamination to measuring environmental variables to counting the MPs in the samples.

Studies seeking to identify MPs in samples also struggle with issues related to classifying particles as plastic or not. Often, it is only possible to chemically identify a subset of potential MPs, but over 50% of these particles might not be plastic (Kroon et al., 2018). Study authors then either adjust their reported concentrations based on the percentage of the subset that was confirmed to be plastic or report both the unadjusted concentrations and the percentages of different particle types in the subset that they verified chemically. We introduce a novel approach to this problem using data from a subset of chemically verified particles to classify individual potential MPs that were not in the subset using a random forest classification model.

## METHODS

### Study area

**FIGURE 1:**
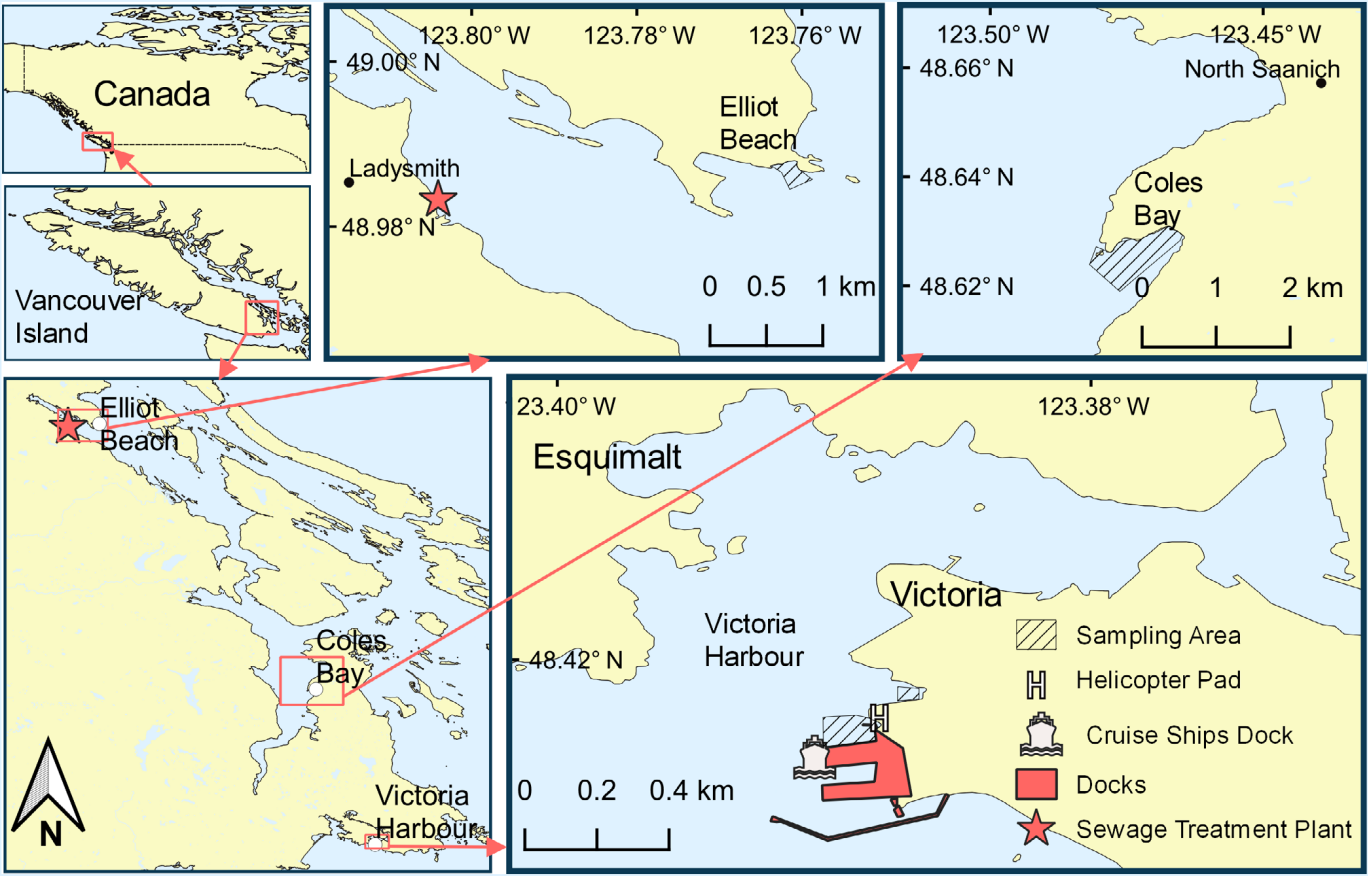
Study areas on Vancouver Island, British Columbia, Canada. The three panels on the right show the three sampling areas, while those on the left show their locations relative to one another and on Vancouver Island. The large breakwater and docks, cruise ship docking area, and nearby commercial helipad of Victoria Harbour's industrial area are indicated.

Samples were collected from three sites on southern Vancouver Island in BC, Canada (Figure 1). Elliot Beach, Coles Bay, and Victoria Harbour were selected as sites because they are representative rural and urban areas with relatively low wave exposure and had similar species available that displayed some degree of residency. Elliot Beach is a rural public beach located at the mouth of a large inlet near the town of Ladysmith. The beach is a mix of exposed bedrock with cobble and sand. Nearby developments are primarily privately owned properties, although toward the head of the inlet, in Ladysmith Harbour, there are several marinas and a large industrial area where many log booms are stored. A secondary sewage treatment plant serving the town of Ladysmith and surrounding area (~17,200 people) is located on the south side of the inlet, across from our sampling area. Coles Bay is a rural, sheltered bay within Saanich Inlet with a wide sandy and cobble, gently sloping beach at its head and bedrock and boulders along its edges. There is a large, predominately subtidal, eel‐grass meadow at the head of the bay. Residential properties and the Pauqachin First Nation reservation are situated on the land surrounding Coles Bay. Victoria Harbour is urban and surrounded by active industrial operations, with our sampling site positioned between a cruise‐ship dock and a commuter helicopter pad, with a large breakwater to the south. The shoreline is highly modified, with little intertidal area, consisting primarily of concrete walls or large boulders.

### Approval for animal experiments

The Animal Care Committee at the University of Victoria approved all fish sample collection and euthanasia procedures according to Canadian Council on Animal Care standards (Protocol No. 2018‐011).

### Water sample collection

Five 1‐L jar samples and five plankton net surface tows were collected at each of the three sites on five separate days during summer low tides in parallel to other sampling efforts (July–August 2018). Both sample types were collected because large‐volume tows can sample a larger spatial area but still underestimate MP concentrations (Covernton, Pearce, et al., 2019). Furthermore, the bulk water samples would represent the MP concentrations present in all sestons, while the plankton tows would be indicative of zooplankton and floating detritus. The jar samples were collected by dipping 1‐L glass mason jars just below the water's surface. The plankton tows were collected using a 150‐μm‐mesh plankton net with a 0.53‐m‐diameter opening. After collection, we used a garden pump sprayer filled with ambient seawater to rinse the net contents into the cod end by spraying the outside of the net. The cod end was then rinsed into a 1‐L glass mason jar using prefiltered deionized water (see sample processing methods). The net was rinsed with seawater between uses. At Coles Bay and Elliot Beach, samples from the shore were collected by wading to a depth of 1 m. The plankton net was towed at the surface parallel to the water's edge for 5 min, with the net held behind and to the side of the person taking the samples to avoid collecting any disturbed sediments. Global Positioning System (GPS) tracking was used to calculate distance traveled and estimate sample volumes based on the size of the circular net opening (~14,781–31,548 L). At Victoria Harbour, there was a steep drop‐off from the shore, so jar samples were collected off the edge of a small dock. The plankton net was towed on the surface by swimming from a point on the dock to the edge of the cruise ship dock (67 m, 67.5 L) while wearing a wetsuit and fins.

### Animal sample collection

Preselected target species were collected across a coastal shallow subtidal and intertidal food web at each site. Where possible, we pretargeted species known to display some degree of site fidelity and would likely have predator–prey interactions with each other. Blue mussels (*Mytilus* spp.), likely a mix of *M. edulis*, *M. trossolus*, *M. galloprovincialis*, and their hybrids in coastal BC (Crego‐Prieto et al., 2015) were hand‐picked from rocks in the upper intertidal. Pacific littleneck clams (*Leukoma staminea*) and Manila clams (*Ruditapes philippinarum*) were dug from mid‐intertidal sand. Small individuals were selected as being potential prey items of local crabs, flatfish, and shiner perch. Clams were secured shut using natural‐rubber elastic bands to prevent gaping, and all bivalve samples were frozen at −20°C following collection. California sea cucumbers (*Apostichopus californicus*) and orange sea cucumbers (*Cucumaria miniata*) were collected from under lower intertidal rocks during low tide via wading or snorkeling. Sea cucumbers were then anesthetized using dilute magnesium chloride (Lewbart & Mosley, 2012), and their digestive tracts were removed and frozen at −20°C. Graceful rock crabs (*Metacarcinus gracilis*), Dungeness crabs (*Metacarcinus magister*), and red rock crabs (*Cancer productus*) were hand‐collected in the shallows via wading during low tide or from the subtidal via scuba. Crabs were euthanized with clove oil (>400 mg L^−1^, according to University of Victoria standard operating procedures), and their stomachs were removed and frozen at −20°C. Leather stars (*Dermasterias imbricata*) were hand‐picked from subtidal rocks during low tide, flash‐frozen at −80°C, and later thawed and their stomachs (pyloric and cardiac) removed. Shiner surfperch (*Cymatogaster aggregata*), starry flounders (*Platichthys stellatus*), and English sole (*Parophrys vetulus*) were caught using a beach seine and euthanized using clove oil (>400 mg L^−1^), and their digestive tracts and livers were removed and frozen at −20°C. Scuba divers captured copper rockfish (*Sebastes caurinus*) and black rockfish (*Sebastes melanops*) via spearfishing and severed their gill arteries quickly with a dive knife. Rockfish digestive tracts and livers were removed and frozen at −20°C. Various body measurements and weights were taken for all animals (Table 1). We aimed to collect at least 10 individuals each from an animal taxon performing a similar ecological function (e.g., flatfish, surfperch, clams, rockfish, sea cucumber). No intertidal beach section was available at Victoria Harbour, so we did not collect clams at this site. Surfperch were collected via beach seine from a boat ramp found around the corner from the cruise‐ship docks at Victoria Harbour, but we were unable to collect enough flatfish at Victoria Harbour or Elliot Beach for MP analysis.

**TABLE 1:** Biometrics and sample size data for animals collected from three sampling sites.

Site	Species	Common name	Tissues analyzed for MPs	Body measurement (mean) range (cm)	Wet body wet (mean) range (g)	Sample size
Coles Bay	*Mytilus* spp.	Blue mussel	Whole body	(1.9) 1.3–2.4 shell length	(0.8) 0.3–1.8	19
	*Leukoma staminea*	Pacific littleneck clam	Whole body	2.1 shell length	2.8	1
	*Ruditapes philippinarum*	Manila clam	Whole body	(2.0) 1.3–2.6 shell length	(2.1) 1.0–3.7	12
	*Apostichopus californicus*	California sea cucumber	Digestive tract	–	(14.3) 2.7–40.9	14
	*Metacarcinus gracilis*	Graceful rock crab	Stomach	(7.0) 6.1–8.1 carapace width	(57.5) 30.1–94.9	16
	*Cymatogaster aggregata*	Shiner surfperch	Digestive tract & liver	(8.5) 7.8–9.2 total length	(8.0) 5.6–9.6	16
	*Dermasterias imbricata*	Leather star	Digestive tract & liver	(12.4) 10.2–15.4 diameter	(60.5) 31.9–120.2	16
	*Parophrys vetulus*	English sole	Digestive tract & liver	(13.5) 11.6–15.6 total length	(26.8) 13.2–50.2	11
	*Platichthys stellatus*	Starry flounder	Digestive tract & liver	(16.0) 14.5–18.3 total length	(38.6) 20.6–66.7	4
	*Sebastes caurinus*	Copper rockfish	Digestive tract & liver	(14.9) 11.3–25.0 total length	(29.2) 21.2–243.2	10
Elliot Beach	*Mytilus* spp.	Blue mussel	Whole body	(1.6) 1.0–2.9 shell length	(0.4) 0.2–0.7	13
	*Ruditapes philippinarum*	Manila clam	Whole body	(1.7) 0.9–2.3 shell length	(1.6) 0.2–3.4	12
	*Cucumeria miniata*	Orange sea cucumber	Digestive tract	–	(22.9) 1.5–54.1	14
	*Apostichopus californicus*	California sea cucumber	Digestive tract	–	4.0	1
	*Metacarcinus magister*	Dungeness crab	Stomach	(8.1) 7.0–9.2 carapace width	(69.3) 45.1–116.5	16
	*Cymatogaster aggregata*	Shiner surfperch	Digestive tract & liver	(9.3) 8.1–10.0 total length	(10.0) 7.0–15.0	16
	*Dermasterias imbricata*	Leather star	Stomachs	(12.8) 10.2–15.6 diameter	(77.7) 37.1–123.7	15
	*Sebastes caurinus*	Copper rockfish	Digestive tract & liver	(21.7) 13.2–31.2 total length	(191.4) 55.8–572.1	18
	*Sebastes melanops*	Black rockfish	Digestive tract & liver	(20.7) 18.5–22.4 total length	(138.9) 110.4–169.4	3
Victoria Harbour	*Mytilus* spp.	Blue mussel	Whole body	(1.2) 0.9–1.6 cm shell length	(0.3) 0.1–0.5	14
	*Cucumaria miniata*	Orange sea cucumber	Digestive tract	–	(36.8) 3.3–96.7	16
	*Cymatogaster aggregata*	Shiner surfperch	Digestive tract & liver	(6.9) 6.1–7.8 total length	(4.7) 2.8–12.8	17
	*Metacarcinus magister*	Dungeness crab	Stomach	(8.3) 7.1–10.8 carapace width	(77.5) 54.8–149.1	12
	*Cancer productus*	Red rock crab	Stomach	(9.1) 8.0–9.9 carapace width	(86.4) 59.5–114.1	3
	*Sebastes caurinus*	Copper rockfish	Digestive tract & liver	(25.6) 19.2–31.2 total length	(294.0) 123.9–479.4	13
	*Sebastes melanops*	Black rockfish	Digestive tract & liver	(20.1) 16.5–22.3 total length	(123.2) 75.2–166.4	8

*Notes*: Shell length was measured for bivalves (longest dimension), carapace width for crabs (longest dimension across carapace), diameter for sea stars (distance between tips of two longest arms), total length of fish (measured from snout to tip of longest lobe of caudal fin). Sea cucumber length was not measured because they can rapidly expand and contract at will. MP = microplastic particle.

### Stable‐isotope analysis

Stable‐isotope analysis was conducted for all animals that were analyzed for MP content. Tissue samples were collected from clam foot muscle, mussel adductor muscle, sea cucumber buccal retractor muscle, sea star body wall (from an arm), crab muscle (from the merus of a cheliped), and fish muscle and liver (enough tissue to fill about a third to a half of a 1.5‐ml microcentrifuge Eppendorf tube). Each sample was dried to constant weight at 40°C (either in Eppendorf tubes or aluminum packets) and then placed in a 1.5‐ml microcentrifuge Eppendorf tube if not already in one. Stainless‐steel milling balls were then added to the tube and the sample ground at 30 Hz for at least 2 min, or until completely homogenized, using a MM400 mixer mill (Retsch, Haan, Germany). For each sample, ~0.5–1.5 mg of dried, homogenized tissue was placed in a tin capsule and crimped shut. To achieve more accurate δ^13^C measurements, inorganic carbon from the sea star ossicles was removed by preparing additional sea star tissue samples in silver capsules and acid fumigating in a desiccator containing a beaker of 12‐M HCl for 8 h (Gale et al., 2013; Gianguzza et al., 2016).

The isotope tissue samples were analyzed for δ^13^C and δ^15^N at the UC Davis Stable Isotope Facility using a PDZ Europa ANCA‐GSL elemental analyzer interfaced to a PDZ Europa 20–20 isotope ratio mass spectrometer (Sercon, Cheshire, UK). The samples were combusted at 1000°C in a reactor packed with chromium oxide and silvered copper oxide, and oxides were removed in a reduction reactor (reduced copper at 650°C). The samples then had N_2_ and CO_2_ separated on a Carbosieve GC column (65°C, 65 ml min^−1^) before entering the isotope ratio mass spectrometer. Ten standards, composed of dogfish muscle tissue samples of consistent isotopic values, were sent to the facility to be processed alongside the experimental samples to quantify variance.

### Quality assurance and control

To reduce sample contamination, laboratory workers wore yellow Tyvek suits (high‐density polyethylene) over their clothing during sample processing, prefiltered all reagents, and deionized water through 1‐μm glass‐fiber filters and conducted all work in an AirClean 600 laminar‐flow hood (AirClean Systems, Creedmoor, NC). No particles from the Tyvek suits were found in any of the samples. All glassware and other equipment that contacted the samples was rinsed at least three times with filtered deionized water, while inside the laminar hood, before use. A Blue Pure 211+ Air Purifier (Blueair, Chicago, IL) always filtered the laboratory air and surfaces were regularly wiped down with 70% ethanol. Three procedural blanks consisting of clean empty glass beakers subjected to the same procedures as the experimental samples were tested alongside each sampling run, for a total of 90 blank samples. After filtration onto polycarbonate (PCTE) membrane filters (Sterlitech Corp., Kent, WA), samples were stored in polystyrene PetriSlides (EMD Millipore, Oakville, ON, Canada).

### Water sample processing

The jar samples were directly filtered onto 1‐μm polycarbonate membrane filters. The plankton tow samples were filtered first through a 4.75‐mm sieve and then rinsed back into the original sample jars. Square 8‐μm nominal stainless‐steel mesh was placed over the jar openings, secured using the rings from the mason jar lids, and the jar inverted onto the mouth of a filter flask and vacuum filtered to remove all water (Covernton, Collicutt, et al., 2019). The jar and plankton tow samples were dried at 40°C to constant weight. Once dry, the plant and algal tissues in the plankton tow samples were digested by adding 200 ml of 30% hydrogen peroxide (H_2_O_2_), sonicating for 5 min, and incubating at 40°C for 48 h (Nuelle et al., 2014). Samples were rinsed with deionized water and filtered again through the stainless‐steel mesh. To remove animal tissues, 100 ml of 10% potassium hydroxide (KOH) were added to each sample, sonicated again for 5 min, and incubated at 40°C for 48 h (Foekema et al., 2013; Thiele et al., 2019). The digestate was again rinsed and filtered to remove KOH and dried at 40°C to constant weight. To remove sand, each sample was rinsed with 100 ml of 52% sodium iodide (NaI), with a density of 1.6 g ml^−1^, into a separatory funnel (Claessens et al., 2013). The funnel was left for at least 1 min before releasing the bottom fraction and rinsing the supernatant through 250‐ and 150‐μm sieves. The two sieved fractions were separately filtered onto 8‐μm polycarbonate membrane filters using a vacuum pump and a six‐port filtration manifold. The filtered NaI solution was reused by adjusting its density back to 1.6 g ml^−1^, using solid NaI, and refiltering (Kedzierski et al., 2017).

### Animal sample processing

The bivalves were defrosted at room temperature, the outside of their shells rinsed, and all the soft tissues removed from the shells. The tissues were placed into small glass beakers covered with aluminum foil and dried at 40°C to constant weight. The tissues were digested by adding 20 ml of 10% KOH to each sample, incubated at 40°C for 5 days, and filtered to 1 μm. After primary filtration and rinsing, any remaining fatty tissue was dissolved by adding 20 ml of sodium dodecyl sulfate to the filter funnel for 5 min, before rinsing and filtering the samples several times. To remove sand, each membrane filter was placed within a clean beaker holding 30 ml NaI solution for a minimum of 15 min, then the filter was removed and carefully rinsed of any adhered particulates using more NaI solution. Density separation was conducted using the same procedure as for the plankton tow samples, and the samples were filtered to 1 μm.

The remaining animal samples were processed using methods similar to those used for the bivalves, with some modifications. The gastrointestinal tracts of sea cucumbers were digested using 30 ml KOH and the digestate sieved into <150‐ and ≥150‐μm fractions prior to density separation, with <150‐μm fractions filtered to 1 and ≥150‐μm fractions to 8 μm. Crab stomachs were digested using 50 ml KOH for 5 days and the digestate sieved into <1‐ and ≥ 1‐mm fractions. The smaller fractions were filtered to 1 μm, as with the bivalve samples, while the larger fractions were rinsed into a petri dish and dried at 40°C prior to particle counting. Sea star stomachs were digested with 50 ml KOH for 5 days and split into <150‐ and ≥150‐μm fractions and filtered, as with the sea cucumbers. For flatfish, surfperch, and rockfish, gastrointestinal tracts (including pyloric caeca) and livers were processed separately using the same protocol as with sea star stomachs, although the livers were not size‐fractioned because the tissues were easily digested with little remaining material. A density separation step (same as described earlier) for the flatfish digestive tract samples was also added, but not for the crab, sea star, surfperch, and rockfish gut samples. For the rockfish, gastrointestinal tracts were cut open using scissors and the contents rinsed through a 4.75‐mm sieve. The larger sieved fraction was made up of partially digested animals, which were photographed, digested, and filtered separately from the filtrate and the rest of the stomach and intestines. The gut animal samples, and the remaining gastrointestinal tract samples, were sieved into ≥1‐mm fractions in addition to <150‐ and ≥150‐μm fractions. For the different sample types described earlier, the samples were split into different size classes based on how much indigestible material was present, in order to optimize filtration.

### Particle analysis

Visual analysis was conducted for all sample types, other than the ≥1‐mm fraction for crabs and rockfish, by placing the PetriSlides on a compound microscope stage, removing the cover, and manually scanning the whole sample at 100× magnification, using both transmitted and reflected light sources that were continually adjusted to optimize particle finding. For the ≥1‐mm fraction crab and rockfish samples, the petri dishes were viewed using a dissecting microscope. To avoid sample contamination during microscopy, the microscope stage was enclosed in a clear plastic bag that was taped to the bench top and microscope at its edges, as per Torre et al. (2016). Any potential MPs were identified during visual scanning, removed with forceps, and placed and labeled on double‐sided sticky tape. Any unnaturally or brightly colored particles were classified as potential MPs, as well as clear fibers that did not look natural (i.e., lacked internal structure, including striations, and were an even width along their length). These criteria were established based on past experience, exchange of information with other researchers, and references such as Greaves and Saville (1995) and Hidalgo‐Ruz et al. (2012). Shape (e.g., fibers, fragments, spherules, films), color, and length (longest dimension) of each potential MP were recorded.

Of the 1124 potential MPs that were counted across all samples, 882 (78%) particles were extracted (without accidentally losing the particle during transfer) and analyzed using a micro‐Raman spectrometer (HORIBA Raman Xplora Plus, HORIBA, Kyoto, Japan). Chemical identification via Raman spectroscopy was carried out using a 785‐nm (range 50–2000 cm^−1^) or 532‐nm (range 50–4000 cm^−1^) laser with a 100× long‐working‐distance microscope objective with a filter ranging from 0.1% to 100%; gratings of 600 or 1200 grooves mm^−1^; 1–15 s acquisition time; 2, 4, 6, 8, or 10 accumulations; a confocal hole diameter of 100 or 300 μm; and a confocal slit width of 50 or 100 μm. When acquiring spectra, parameters were optimized, including adding delay time, to inhibit poor resolution, fluorescence, and particle burning. Spectra were compared to the SLoPP and SLoPP‐E libraries (Munno et al., 2020), as well as to the Wiley KnowItAll Raman Spectral Library. Of these 882 particles, 779 (88% of analyzed, or 69% of total particles) were successfully identified and categorized as synthetic—e.g., polyester, nylon, polyurethane, acrylic—or natural, including semisynthetic (rayon), environmental (e.g., clear cellulosic fibers, minerals, salt), and natural anthropogenic (dyed cellulosic fibers, wool) particles. The remaining particles could not be classified due to poor spectra quality, e.g., as a result of burning or fluorescence or a strong dye signature that could not be confidently associated with a material type.

### Data analysis

All analyses were carried out using R version 4.1.2 (R Core Team, 2021). Data on particle color, shape, and material composition are presented using raw data that are not blank corrected (as this was not done according to color or polymer). For modeling purposes, the 345 particles of unknown identity had to be assigned to the synthetic or natural categories. To do this, we applied a random forest classification model using the Randomforest package (Liaw & Wiener, 2002). The model was specified with particle type (synthetic or natural) as the response variable and expert user ID, sample type (e.g., blanks, plankton tow, mussels), particle size, shape, color, and length as predictor variables. User ID reflected expert opinion (in this case GAC) as to whether a particle was natural or synthetic in composition, depending on factors including fiber structure and shape (based on hundreds of hours of visual and chemical analyses of potential MPs). The model was run over 1000 trees with two variables allowed per split. After classification, we calculated total synthetic particle counts (MPs) for each sample, pooling across size fractions.

Bayesian, generalized linear mixed‐effects models (GLMMs) were used to explore several aspects of the data using JAGS (Plummer, 2003), implemented via the R2jags (Su & Yajima, 2020) package. The structure of each model was determined to explore the relationship between trophic position and MP concentration, while including all relevant covariates such as species and site that could feasibly have influenced these concentrations as well. There were only three sites, so this variable was included as a fixed effect, where different local environmental MP concentrations might shift the intercept of the relationship with trophic position. Species was treated as a random effect, where some consistent effect of trophic position might be expected, with species traits causing realized MP concentrations in digestive tracts or livers to be distributed around the mean. Poisson distributions were used because the MP data in the samples and procedural blanks were positive integer counts and because two Poisson distributions could be added together to create a third, allowing us to simply deal with background contamination. Model fits were assessed using the DHARMa package (Hartig, 2020), and, if the model's simulated scaled‐residuals plots suggested misspecification, the model structure was tweaked until the issue was resolved, where possible (Bolker et al., 2009). Three Markov chain Monte Carlo (MCMC) chains were run for each model. When fitting models, the number of MCMC iterations was increased until $\hat{R}$ values, a standard convergence metric, for each estimated parameter reached 1.01 or lower. Hierarchical model structures were used for each GLMM, which accounted for uncertainty at several different levels.

In the lowest layer, we calculated the isotopic value for the baseline consumer, $\delta^{15}N_{\text{base}}$, at each site using the δ^15^N values from mussels. Bivalves make excellent baseline references for isotopic modeling because they are sedentary and consume a relatively consistent diet (Anderson & Cabana, 2007). To build uncertainty into $\delta^{15}N_{\text{base}}$ a prior distribution of $\delta^{15}N_{\text{base}}∼\text{Gamma}\left(\alpha ,\beta \right)$ was specified, where mussel isotopic values from a site are assumed to be drawn from a gamma distribution with shape parameter α and rate parameter β. The gamma distribution has mean μ=α/β and variance, $\sigma^{2}=\alpha /\beta^{2}$, which can be solved so that $\alpha =\mu^{2}/\sigma^{2}\;$ and $\beta =\mu /\sigma^{2}$. Thus, the prior distribution for $\delta^{15}N_{\text{base}}\;$ for each site can be specified using the mean, μ, and standard deviation, σ, of mussel δ^15^N from each site.

In the second layer, the trophic position for an individual, ${\mathrm{TP}}_{i}$, was approximated using a rescaled estimate according to Hussey et al. (2014), which accounts for a linear change in the δ^15^N trophic discrimination factor with increasing trophic level. They proposed the equation

$${\mathrm{TP}}_{i}=\frac{\log\left(\delta^{15}N_{\lim}-\delta^{15}N_{\text{base}}\right)-\log\left(\delta^{15}N_{\lim}-\delta^{15}N_{i}\right)}{k}+2$$

where $\delta^{15}N_{\lim}$ is the saturating isotope limit as the trophic position increases, $\delta^{15}N_{\text{base}}$ is the isotopic value of the baseline consumer (trophic level 2) in the food web, $\delta^{15}N_{i}$ is the isotope value of the sampled individual, and k is the rate at which $\delta^{15}N_{\mathrm{TP}}$ approaches $\delta^{15}N_{\lim}$. We used values of 0.315 for k and 21.926 for $\delta^{15}N_{\lim}$ based on the results of their meta‐analysis. The liver δ^15^N values were less enriched, on average, than the muscle values for fish, so we used the average of the two for $\delta^{15}N_{i}$. Uncertainty was also built into $\delta^{15}N_{i}$ using the prior, $\delta^{15}N_{\mathrm{TP}}\approx \text{Normal}\left(\delta^{15}N_{\text{sample}},0.052\right)$, where $\delta^{15}N_{\text{sample}}$ is the isotopic value of a sample, and 0.052 is the standard deviation of the dogfish muscle standards that were analyzed alongside the stable‐isotope tissue samples to quantify methodological variance.

To include uncertainty about observed particle counts in samples that might have been exposed to background contamination in the laboratory, we used a novel, probabilistic method that built a correction into the third layer of the hierarchical GLMMs. The Poisson distribution approximated the observed particle count data well because it was not highly dispersed. We assumed that observed particles $P_{\text{observed}}$ were Poisson distributed with mean $\lambda_{\text{observed}}$ and that the observed mean particle counts were equal to the “true” mean of the sample counts $\lambda_{\text{sample}}$ plus the mean contamination entering a sample during processing, which was approximated by the mean MP count for the blank samples run alongside each sample. This is denoted by

$$P_{\text{observed}}∼\text{Poisson}\left(\lambda_{\text{observed}}\right)$$

$$\lambda_{\text{observed}}=\lambda_{\text{sample}}+\lambda_{\text{blanks}}$$

$$\log\left(\lambda_{\text{sample}}\right)=L_{j}$$

with $L_{j}$ being the linear equation for whichever model relating some predictor variables to the concentration of particles in the sample.

Several GLMMs were run on different portions of the data according to different linear equations. Each model had the previously described layers of uncertainty structure built in, if relevant. To explore differences in seawater MP concentrations among sites, the linear equation $L_{\text{seawater}}=\alpha_{\text{site}}$ was applied, with $\alpha_{\text{site}}$ the intercept term, according to the site, with prior $\alpha_{\text{site}}\approx \text{Normal}\left(0,10\right)$. To account for volume in the plankton tows, an offset term for sample volume *V* was added to the equation so that $L_{\text{seawater}}=\log\left(V\right)+\alpha_{\text{site}}$. For the plankton tow model, MCMC chains were run for 10,000 iterations with a burn‐in of 500 and a thinning factor of 5. For the jar‐collected seawater samples, 2000 iterations were used, with a burn‐in of 500, and without thinning. For both models, posterior predictive samples were generated for each site.

The relationship between trophic level and number of particles in the digestive tracts of animals (whole bodies for bivalves) was modeled according to $L_{\text{digestive tracts}}=\alpha_{\text{species}}+\beta_{\mathrm{TP}}{\mathrm{TP}}_{i}+\gamma_{\text{site}}$, with $\alpha_{\text{species}}$ a random intercept according to the sample species defined as $\alpha_{\text{species}}\approx \text{Normal}\left(0,\sigma_{\text{species}}\right)$ with prior $\sigma_{\text{species}}\approx \text{Exponential}\left(1\right)$, $\beta_{\mathrm{TP}}$ a random slope for trophic position according to the site with prior $\beta_{\mathrm{TP}}\approx \text{Normal}\left(0,1\right)$, and $\gamma_{\text{site}}$ a fixed effect of the site with prior $\gamma_{\text{site}}\approx \text{Normal}\left(0,1\right)$. Note that more regularizing priors were used relative to the water samples because of the much larger sample size. The MCMC chains were run over 7000 iterations, with a burn‐in of 500 and a thinning factor of 2. Individual‐level bioaccumulation factors were calculated according to $\mathrm{BAF}=C_{a}/C_{w}$, where $C_{a}\;$ is the MP concentration in the digestive tract of the animal, calculated by dividing the mean of the posterior for animal $\lambda_{\text{observed}}$ by the total wet body weight (kg) of the animal, and $C_{w}$ is the mean of the posterior for seawater $\lambda_{\text{observed}}$ in particles per liter from the jar samples at each site. We used the jar samples here because the concentrations were much higher than the net tow sample estimates and likely more representative of true seawater concentrations (Covernton, Pearce, et al., 2019). To quantify the effect of trophic level on digestive tract MP concentrations in animals, posterior predictive simulations were generated from the model over 2000 randomized combinations of all sites, species, and trophic position values ranging from one to six. Predictions were generated at the site level and combined across species. To quantify differences by species, posterior predictive simulations were also generated holding the estimate of trophic position for each species at its mean value and combining effects across site.

The relationship between trophic level and number of particles per gram of wet tissue in the fish livers was modeled according to $L_{\text{livers}}=\log\left(W_{\text{sample}}\right)+\alpha_{\text{species}}+\beta_{\mathrm{TP}}{\mathrm{TP}}_{i}+\gamma_{\text{site}}$, with $W_{\text{sample}}$ the wet weight (ww) of the sample, and the other terms and priors identical to the previous model. The MCMC chains were run over 5000 iterations, with a burn‐in of 500 and no thinning. To quantify the degree of biomagnification in fish livers, trophic magnification factors were calculated using the posterior estimate for slope according to trophic position as $\mathrm{TMF}=e^{\beta_{\mathrm{TP}}}$. To quantify the effect of trophic level on fish liver MP concentrations, posterior predictive simulations were generated from the model with over 2000 randomized combinations of all site and trophic‐position values ranging from 1 to 4.5. Means and credibility intervals for the posterior estimates were generated at the site level.

To quantify trophic transfer in the rockfish digestive tract data, the number of particles present in the partially digested material, in terms of particles per gram of dry weight (dw) according to site, was modeled using the linear equation $L_{\text{transfer}}=\log\left(W_{\text{sample}}\right)+\alpha_{\text{species}}+\gamma_{\text{site}}$ with the same priors as used earlier. The MCMC chains were run over 2000 iterations, with a burn‐in of 500 and no thinning. To estimate the mean concentration of MPs expected to be transferred from ingested animals, posterior predictive samples were generated—combining the effects of all species and sites.

Assuming that the rockfish were all feeding prior to capture, comparing the difference in digestive tracts for individuals with full and empty stomachs should offer insight into whether most MPs were excreted alongside ingested food. We modeled this using the linear equation $L_{\text{rockfish guts}}=\alpha_{\mathrm{gut}}+\gamma_{\text{site}}+\gamma_{\text{species}}$, where $\alpha_{\mathrm{gut}}$ is a fixed effect according to whether a rockfish had a full or empty gut, determined by whether or not a stomach contained any ingested animals on the 1‐mm sieve, with prior $\gamma_{\mathrm{gut}}\approx \text{Normal}\left(0,1\right)$ and the other parameters and priors the same as previously mentioned. There were some slight issues with heterogeneity in the model fit that could not be resolved due to a higher variance in MP counts in the full guts. The MCMC chains were run for 5000 iterations, with a burn‐in of 500 and no thinning. Posterior predictive samples were generated across all combinations of species, site, empty or full stomachs, and 1000 values of trophic position and total length, ranging from the minimum to maximum values in the data set. Overall predicted means and credibility intervals of posterior estimates were then calculated at the level of species and stomach fullness.

## RESULTS

### Stable‐isotope analysis

**FIGURE 2:**
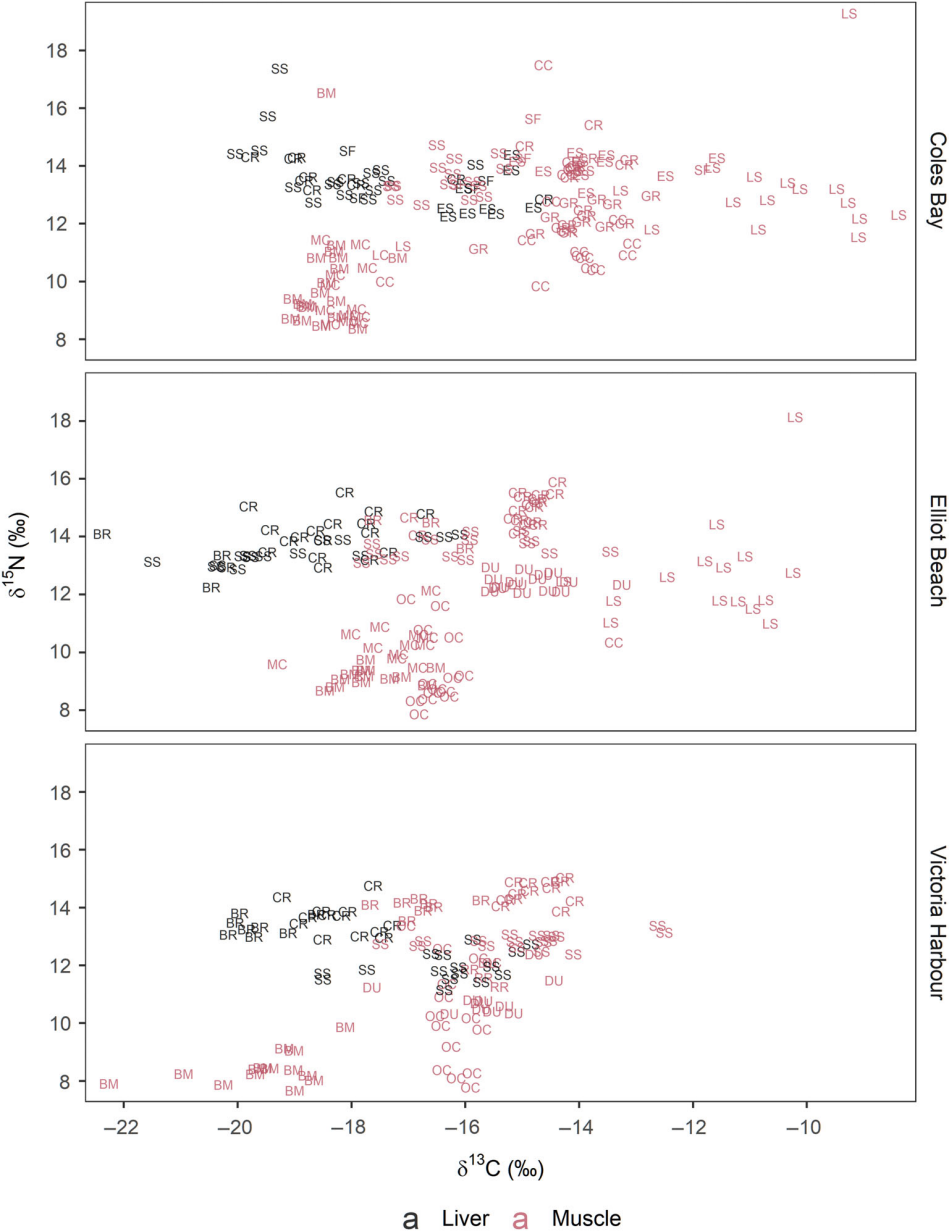
Stable carbon and nitrogen isotopic biplot for species collected at the three sample sites. Both liver and muscle samples were collected for the fish, and tissue types are separated by color. Species are labeled by abbreviated common names (BM, blue mussel; LC, littleneck clam; MC, Manila clam; OC, orange sea cucumber; CC, California Sea cucumber; LS, leather star; RR, red rock crab; GR, graceful rock crab; DU, Dungeness crab; SS, shiner surfperch; ES, English sole; SF, starry flounder; CR, copper rockfish; BR, black rockfish).

Mussels and clams had similar δ^13^C values, with most individuals from the other species more enriched in ^13^C (Figure 2). Sea stars were particularly enriched in ^13^C relative to the other taxa. Similar δ^15^N values occurred among individuals within a given species, other than mussels, clams, sea cucumbers, and sea stars, which displayed relatively large variation in δ^15^N. Two individual sea stars, one from Coles Bay and one from Elliot Beach, had δ^15^N values higher than any other animal.

### Particle analysis

The procedural blanks contained means of 0.24 ± 0.57 (±SD) synthetic and 0.50 ± 0.97 natural particles. Of the 1124 potential MPs counted, including from blanks, 97.9% were fibers, 1.9% were fragments, and 0.2% were films (see Appendix S1: Figures S1 and S2 for a breakdown across sample types). The most common particle colors were clear (26.9%), blue (24.0%), and black (22.0%) (Appendix S1: Figure S1). For the 779 particles identifiable with Raman spectroscopy, 32.5% were classified as synthetic and 67.5% as natural, which could be further broken down as 10.4% natural environmental (e.g., bone, mineral, clear cellulose), 54.0% natural anthropogenic (e.g., wool, dyed cellulose), and 3.1% semisynthetic (e.g., rayon) (Appendix S1: Figure S2). Overall, polyester fibers were the most abundant type of MP found in the samples (80.2% of synthetic particles). In the synthetic category for Raman‐identified particles, the blanks contained only black, clear, and blue fibers—all polyester except for one clear polyacrylonitrile fiber—as well as one multicolored polystyrene fragment. In the environmental samples, 63.3% of synthetic, Raman‐identified particles matched these characteristics. The random forest model displayed a sensitivity of 75.1% and specificity of 81.0% for classifying particles as synthetic or not, with an overall accuracy of 79.1%, during fitting (Appendix S1: Figure S3). According to a variable importance plot, the strongest predictor of particle type was user ID.

### Seawater concentrations

**FIGURE 3:**
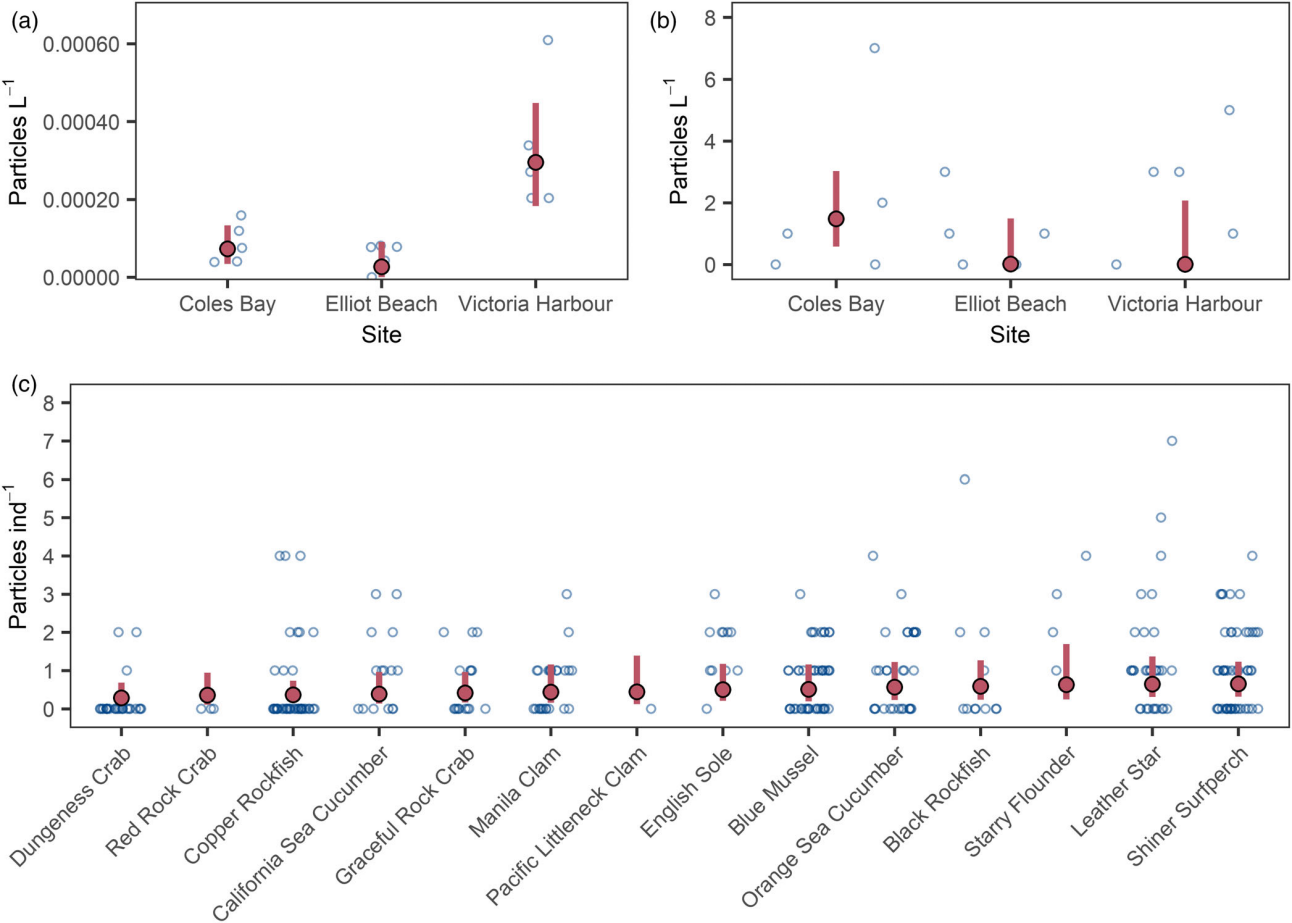
Microplastic particle concentration from (a) plankton tow water samples, (b) jar water samples, and (c) digestive tracts by species. The open circles are raw, uncorrected counts. The red circles and lines indicate mean posterior predictive mean concentrations and 95% credibility intervals for simulated model predictions, respectively. Animal species are arranged from left to right by increasing mean predicted digestive tract microplastic particle concentration. For the animal generalized linear mixed‐effects model, the simulations were generated using the average trophic position for each species and the mean and credibility intervals calculated from joint Markov chain Monte Carlo posterior samples across the three sites.

After classifying unknown particles, the uncorrected MP concentrations estimated by the plankton tows were 8.64 × 10^−5^ ± 5.19 × 10^−5^ particles L^−1^ (mean ± SD) for Coles Bay, 5.59 × 10^−5^ ± 3.49 × 10^−5^ particles L^−1^ for Elliot Beach, and 3.25 × 10^−4^ ± 1.68 × 10^−4^ particles L^−1^ for Victoria Harbour. We did not see any particles in plankton tow samples that matched the waders or wetsuit worn during sampling. Taking background contamination into account, the corrected GLMM estimated mean particle concentrations to be 7.26 × 10^−5^(3.41 × 10^−5^ to 1.33 × 10^−4^, 95% credibility interval) particles L^−1^ for Coles Bay, 2.67 × 10^−5^ (2.29 × 10^−7^ to 9.04 × 10^−5^) for Elliot Beach, and 2.95 × 10^−4^ (1.82 × 10^−4^ to 4.47 × 10^−4^) for Victoria Harbour (Figure 3a). The posterior for Victoria Harbour did not overlap with Coles Bay or Elliot Beach posteriors (which were similar to one another), indicating a statistically higher MP concentration at this site (Appendix S1: Figure S4). For the 1‐L jar samples, the uncorrected concentrations were 2.00 ± 2.92, 1.00 ± 1.22, and 2.40 ± 1.95 particles L^−1^ for Coles Bay, Elliot Beach, and Victoria Harbour, respectively. The model estimates were 1.48 (0.58–3.03), 0.02 (0.00–1.49), and 0.01 (0.00–2.07) particles L^−1^ for Coles Bay, Elliot Beach, and Victoria Harbour, respectively (Figure 3b). The jar sample posteriors overlapped at all sites, suggesting no substantial statistical differences, although Coles Bay MP concentrations were higher (Appendix S1: Figure S5).

### Digestive tract concentrations and bioaccumulation factors

Uncorrected MP concentrations in animal digestive tracts ranged from 0 to 7 particles per individual (ind^−1^) with a mean of 0.86. The GLMM random intercept posteriors largely overlapped each other, suggesting no substantial differences among species, although there was some separation between Dungeness crabs (lower MP concentration) and shiner surfperch and leather stars (higher MP concentrations; Appendix S1: Figure S6). Simulating from the model while holding trophic position at the average for each species resulted in overall lowest MP concentration for Dungeness crabs of 0.29 (0.11–0.68) particles ind^−1^ and overall highest concentrations for shiner surfperch (0.65, 0.31–1.23 particles ind^−1^) and leather stars (0.64, 0.30–1.36 particles ind^−1^; Figure 3c). The Victoria Harbour animals site coefficient was 3.8 times higher than the site coefficient for Elliot Beach (posterior mean of 1.24 vs. 0.33 MPs ind^−1^) and the Coles Bay site coefficient posterior intermediate (0.65 MPs ind^−1^) and overlapped with the other two sites (Appendix S1: Figure S6).

**FIGURE 4:**
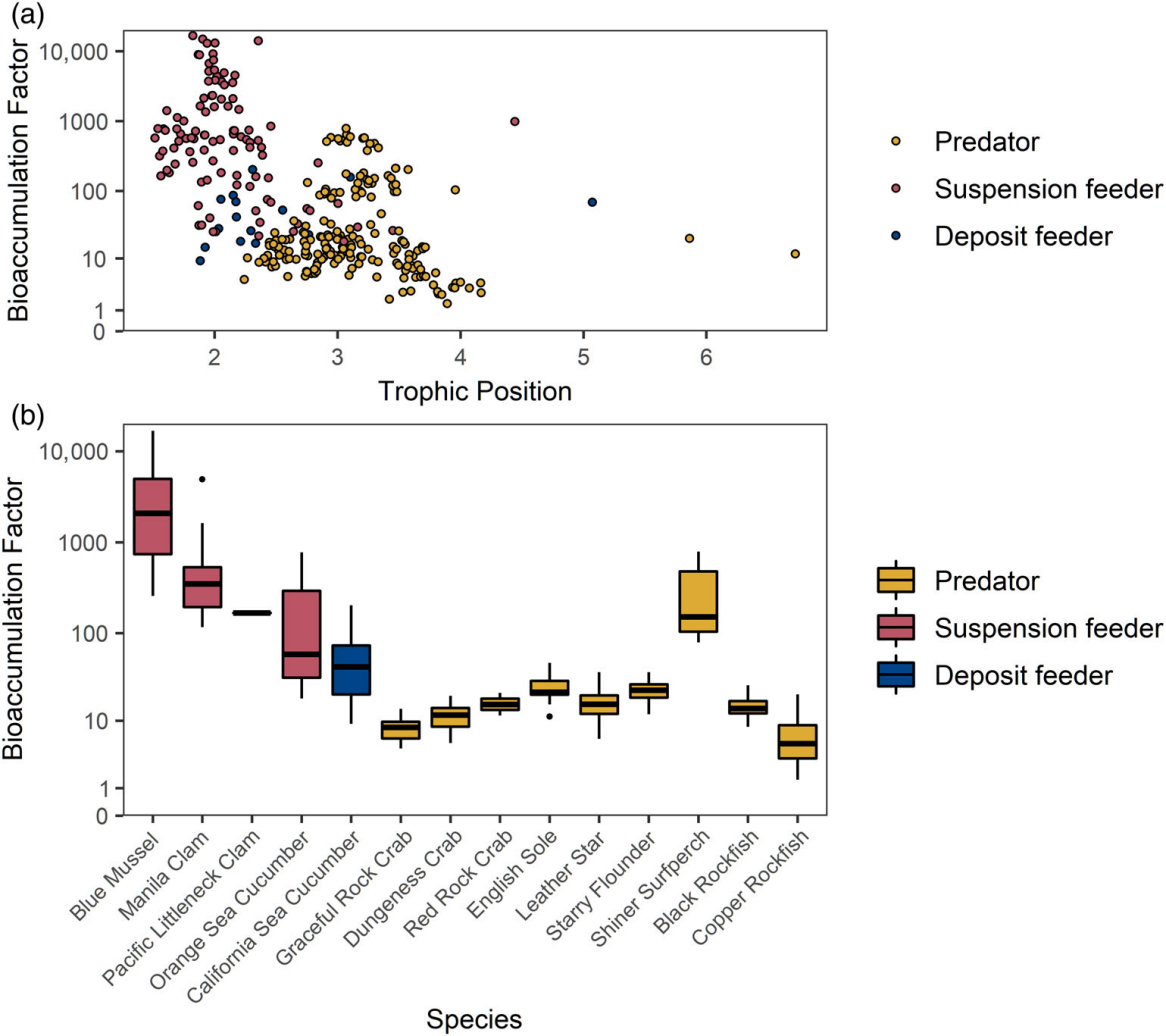
Individual bioaccumulation factors according to (a) trophic position and (b) the same data separated by species (arranged by increasing mean trophic position from left to right). The points and boxplots are colored according to the feeding strategy of each species.

Expressing the mean of the fitted model posterior predictive MCMC samples in terms of the ww and dw of digestive tracts (whole bodies for bivalves) resulted in the highest overall mean concentration estimates for the California sea cucumbers from Elliot Beach and the mussels from Victoria Harbour (Table 2). The lowest mean concentration estimates by tissue weight were for black and copper rockfish from Elliot Beach and Victoria Harbour and leather stars from Elliot Beach (Table 2). Individual bioaccumulation factors decreased exponentially with increasing trophic position (Figure 4a). Shiner surfperch bioaccumulation factors deviated from the overall trend and were higher than might otherwise be expected according to their trophic position (Figure 4b).

**TABLE 2:** Trophic position and digestive tract microplastic concentration estimates for each species in terms of individual (ind), wet weight (ww), and dry weight (dw) of the digestive tract.

Species	Site	Trophic position		Particles ind^−1^		Particles g^−1^ ww		Particles g^−1^ dw	
		Mean	Range	Mean	Range	Mean	Range	Mean	Range
Manila clam	Coles Bay	1.82	1.55–2.34	0.67	0.65–0.71	1.03	0.56–1.79	8.75	3.75–25.73
Blue mussel	Coles Bay	1.98	1.51–4.44	0.78	0.74–1.00	4.14	0.71–9.15	105.99	0.84–1039.74
Littleneck clam	Coles Bay	2.18	…	0.76	…	0.96	…	9.16	…
California sea cucumber	Coles Bay	2.48	1.88–5.07	0.66	0.61–0.88	10.52	0.21–127.56	14.44	1.44–50.16
Graceful rock crab	Coles Bay	2.56	2.24–2.83	0.70	0.69–0.73	1.07	0.36–2.22	6.39	1.47–17.13
Leather star	Coles Bay	2.98	2.27–6.72	1.22	1.13–1.92	0.90	0.18–6.40	5.29	1.29–30.17
English sole	Coles Bay	3.03	2.74–3.36	0.95	0.92–1.00	1.20	0.64–3.31	6.07	2.93–21.23
Shiner surfperch	Coles Bay	3.13	2.76–3.96	1.25	1.22–1.35	3.39	1.31–5.01	25.22	2.88–50.48
Starry flounder	Coles Bay	3.19	2.89–3.46	1.26	1.24–1.31	0.96	0.58–1.50	4.83	3.21–6.68
Copper rockfish	Coles Bay	3.20	3.01–3.60	0.73	0.71–0.75	0.36	0.04–0.74	1.46	0.27–3.91
Blue mussel	Elliot Beach	2.00	1.88–2.15	0.36	0.34–0.35	2.83	1.09–7.12	25.14	7.93–60.13
Orange sea cucumber	Elliot Beach	2.07	1.69–2.75	0.40	0.39–0.41	1.46	0.19–6.20	8.80	0.86–31.98
California sea cucumber	Elliot Beach	2.31	…	0.28	…	17.27	…	1126.15	…
Manila clam	Elliot Beach	2.32	2.08–2.84	0.31	0.30–0.31	1.26	0.28–6.50	12.88	1.84–60.05
Dungeness crab	Elliot Beach	2.94	2.82–3.12	0.22	0.21–0.22	0.28	0.09–0.49	1.83	0.27–4.75
Leather star	Elliot Beach	3.11	2.49–5.86	0.50	0.47–0.63	0.12	0.05–0.24	0.82	0.30–2.31
Shiner surfperch	Elliot Beach	3.34	3.17–3.57	0.52	0.50–0.53	1.45	0.82–2.63	9.86	4.04–24.32
Black rockfish	Elliot Beach	3.41	3.11–3.65	0.50	0.49–0.51	0.11	0.09–0.13	0.55	0.46–0.68
Copper rockfish	Elliot Beach	3.72	3.32–4.17	0.30	0.29–0.31	0.04	0.01–0.09	0.32	0.03–1.16
Blue mussel	Victoria Harbour	2.00	1.82–2.35	0.81	0.75–0.85	14.28	5.62–28.14	403.42	54.90–4047.18
Orange sea Cucumber	Victoria Harbour	2.47	1.85–3.45	0.84	0.67–0.99	0.59	0.06–2.15	4.26	0.75–12.58
Dungeness crab	Victoria Harbour	2.67	2.48–3.10	0.43	0.40–0.45	0.35	0.12–0.68	1.87	0.48–5.51
Red rock crab	Victoria Harbour	2.84	2.75–2.93	0.54	0.52–0.55	0.51	0.46–0.57	2.95	2.46–3.64
Shiner surfperch	Victoria Harbour	3.12	2.91–3.33	0.92	0.88–0.97	3.94	2.41–12.80	23.21	16.55–39.61
Black rockfish	Victoria Harbour	3.57	3.48–3.71	0.80	0.79–0.83	0.11	0.07–0.26	0.67	0.43–0.93
Copper rockfish	Victoria Harbour	3.72	3.5–4.07	0.47	0.43–0.50	0.05	0.02–0.08	0.35	0.15–0.90

*Notes*: Means of posterior predictive samples for trophic position and “true” microplastic particle concentration were used to calculate means and ranges for individuals of each species at each site. Entries are arranged from lowest to highest mean trophic position, within site.

**FIGURE 5:**
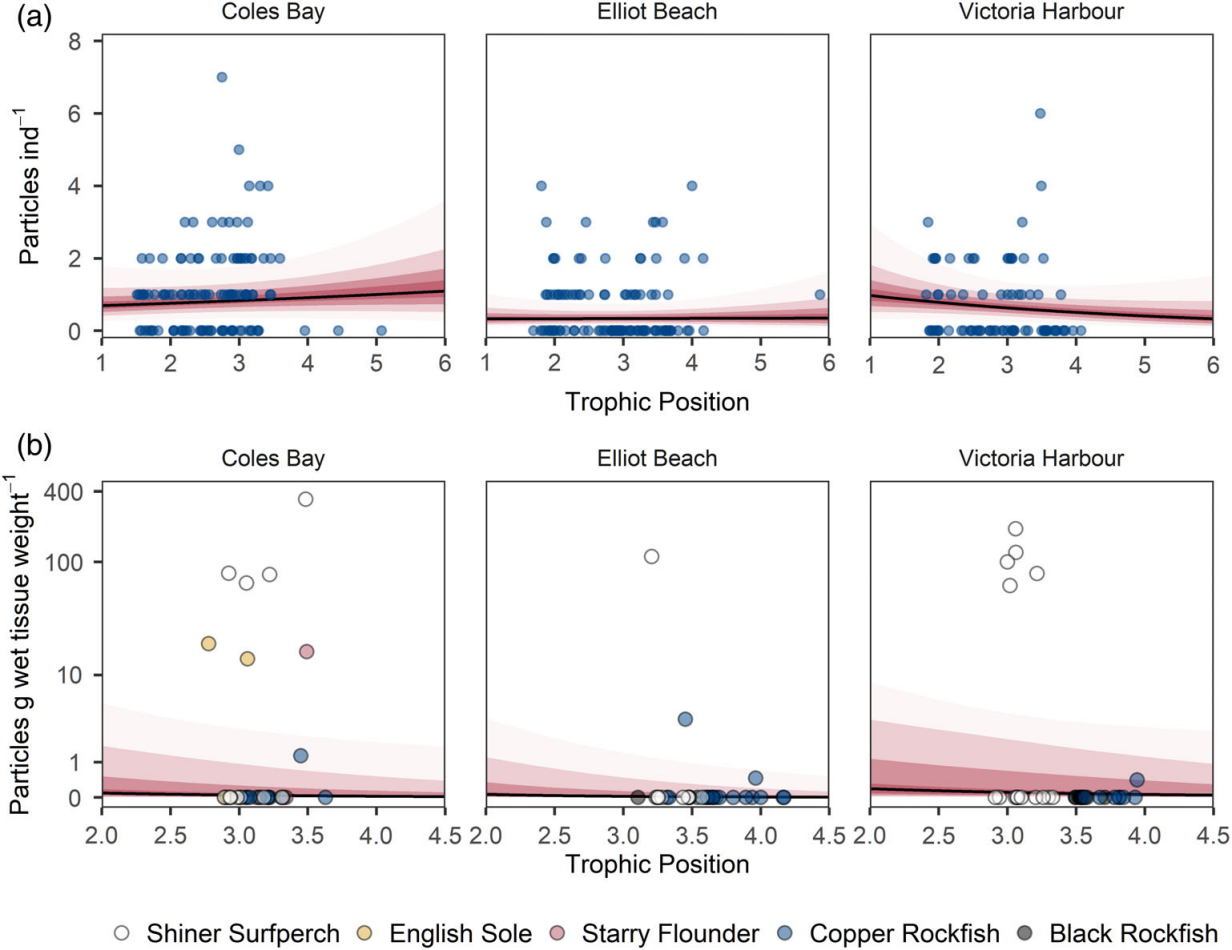
Microplastic particle concentration at three sampling sites in relation to trophic position for (a) digestive tracts of all sampled animals and (b) fish livers. The points represent raw, uncorrected data. In (b) the color of the points denotes fish species. The lines show the mean of posterior predictive Markov chain Monte Carlo samples for Bayesian generalized linear mixed‐effects models. The increasingly light ribbons show 25%, 50%, 75%, and 95% credibility intervals. In (a), the means and credibility intervals were calculated by combining posterior predictive samples across all levels of the species random effect.

There was no correlation between trophic position and MP concentration for animal digestive tracts at either Coles Bay or Elliot Beach and a slightly negative slope for Victoria Harbour (Appendix S1: Figure S6; Figure 5a). The digestive tract concentrations, pooled across species, were lowest for Elliot Beach and highest at Victoria Harbour, with Coles Bay intermediate, but the posteriors for all sites overlapped each other, and so any differences were not large (Appendix S1: Figure S6). Trophic position posterior estimates from the GLMM had a higher spread for Coles Bay because of the higher variance in baseline δ^15^N values compared with other sites (Appendix S1: Figure S7). Coles Bay had the broadest range in trophic levels due to a single leather star with an estimated trophic position of 6.7, whereas Victoria Harbour had the lowest range in trophic levels (Table 2).

### Fish liver concentrations and trophic magnification factor

Uncorrected MP concentrations in the fish livers ranged from 0 to 16.78 particles g^−1^ ww, with a mean of 1.42, across species and sites. However, after correcting for background contamination in the GLMM the estimates were much lower. Simulating from the model across trophic positions ranging from 2 to 4.5 produced mean posterior predictive values for mean MP concentrations ranging from 0.00 to 0.19 particles g^−1^ ww (Figure 5b). The three sites did not differ substantially according to model posteriors, but there was a negative correlation between trophic position and MP liver concentration, especially for Elliot Beach (Appendix S1: Figure S8). However, the model simulation also showed that the difference was <1 particle g^−1^ across trophic levels, with most predicted mean values <1 particle g^−1^ (Figure 5b). The trophic magnification factor—calculated using the posterior estimates for the slope of the relationship between trophic position and MP particles g^−1^ dry tissue weight across all sites and ignoring the effect of species—was 0.53 (0.17–1.20).

### Trophic transfer in rockfish

**FIGURE 6:**
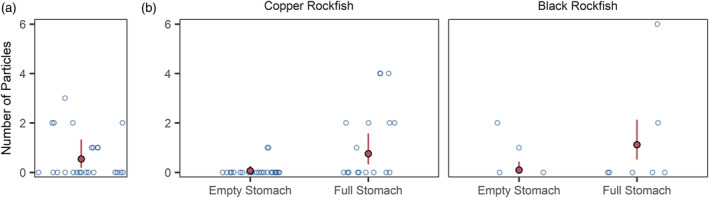
Microplastic concentrations in (a) intact animals from all rockfish stomachs from all species and sites and (b) in rockfish digestive tracts separated by species and stomach fullness. The open circles represent raw, uncorrected data. The filled points show the mean of posterior predictive Markov chain Monte Carlo samples for Bayesian generalized linear mixed‐effects models and are horizontally jittered to improve visibility. The vertical lines show 95% credibility intervals.

The ingested animals separated from the rockfish stomachs had an uncorrected mean MP concentration of 0.67 ± 0.92 particles per sample (pooled for each stomach). The GLMM estimate was a mean of 0.55 (0.19–1.33) particles per sample based on the combined site and species posteriors (Figure 6a). There were no differences by rockfish species or by site (Appendix S1: Figure S9). According to the GLMM exploring differences between rockfish with empty and full stomachs, there were no substantial differences in model posteriors between species or by site, but there was a strong effect of stomach fullness, with the separation of model posteriors for empty versus full stomachs (Appendix S1: Figure S10). Digestive tracts from rockfish with full stomachs held an estimated mean MP concentration of 0.76 (0.32–1.57) and 1.12 (0.52–2.13) particles ind^−1^ for copper rockfish and black rockfish, respectively, compared with 0.07 (0.01–0.26) and 0.10 (0.01–0.44) particles ind^−1^ for the digestive tracts of rockfish with empty stomachs (Figure 6b).

## DISCUSSION

Animals spanning a range of trophic levels, collected from three coastal marine food webs in BC, had low concentrations of MPs >100‐μm in their digestive tracts (<1 particle ind^−1^ on average after accounting for background contamination), with no evidence of biomagnification. Furthermore, calculated bioaccumulation factors decreased with increasing trophic level. More MPs were present in animals at Victoria Harbour compared with the other sites. This was driven by lower trophic animals, as indicated by a negative slope at this site for the relationship between trophic level and MP concentration. Higher MP concentrations were also found at this site by the plankton tow samples, although not by the jar samples, but the jar sample estimates were four orders of magnitude higher than the tow sample estimates. Clear evidence of trophic transfer occurred in the rockfish stomachs. However, low MP concentrations in the digestive tracts of individuals with empty stomachs compared with those containing ingested animals suggest that excretion of MPs is rapid and that accumulation is primarily dependent on the MP ingestion rates relative to body size. MP concentrations in fish livers were also low (0.01–2.66 particles g^‐1^ wet tissue weight after accounting for background contamination), with a trophic magnification factor <1, suggesting trophic dilution (i.e., rather than magnification).

### Trophic position and feeding habits

The trophic position estimates matched well with literature descriptions of the feeding habits of the sampled species. Most of the mobile species are known to display a high degree of site fidelity at the selected body sizes, the exceptions being the shiner surfperch and starry flounders (i.e., no site fidelity) and the copper and black rockfish (intermediate fidelity and a high degree of vertical migration; Day, 1976; Diamond & Hankin, 1985; Gordon, 1963; Hannah & Rankin, 2011; Hildenbrand et al., 2011; Moser et al., 2013; Stone & O'Clair, 2002). It thus seems likely that most of the sampled animals occurred within the same food webs and that the stable‐isotope analysis accurately captured the presumed trophic positions of studied individuals. However, some caution in interpretation might be necessary. The leather stars could present an exception since their δ^13^C values were more enriched than the other species, whereas the δ^13^C values for the other species overlapped sufficiently, suggesting that they depended on similar carbon sources.

Clams and mussels had similar trophic positions and could be assumed to be feeding primarily on phytoplankton and other planktonic particles. The sea cucumber species were slightly higher in trophic position, indicating some consumption of animal materials. Orange sea cucumbers live in crevices beneath rocks and boulders, from where they extend their buccal tentacles for suspension feeding. In contrast, California sea cucumbers live exposed on the substrate and sweep the surface with their feeding tentacles to collect particulate matter (Cameron & Fankboner, 1984). This is reflected in the isotopic results, with California sea cucumbers more enriched in ^13^C than the orange sea cucumbers. Higher trophic position estimates for some California sea cucumber individuals (range of 1.88–5.07) likely reflect ingestion of decomposing animal material, including feces, from higher‐trophic‐level animals.

The size ranges of the crab species considered feed on a wide range of benthos and sometimes juvenile fish and conspecifics (Stevens et al., 1982). These isotopic data supported a greater contribution of animal material to the diet than sea cucumbers, but with some reliance on algal and plant material (especially for graceful rock crabs). Leather stars feed on a wide range of benthic species, including sea anemones, sea cucumbers, hydroids, bryozoans, crab molts, and barnacles (Annett & Pierotti, 1984). This is well reflected by the variable trophic position estimates for this species, suggesting a mix of algal/plant and animal dietary items. The high (>5) trophic position estimates for two individuals suggest that they might have been feeding on the decomposing bodies or feces of high‐trophic‐level animals. The separation between δ^13^C values for the leather stars and bivalves suggests that they may have different base carbon sources, potentially more ^13^C‐enriched benthic producers for the former compared with more depleted primary producers for the latter (Christianen et al., 2017). The crab δ_13_C values were intermediate between bivalves and leather stars, suggesting a mix of benthic and pelagic carbon sources.

Shiner surfperch feed on benthic and pelagic pericarid crustaceans, polychaetes, and copepods, as well as algae, barnacles, and mussels (Barry et al., 1996; Barry & Cailliet, 1981; Gordon, 1963). Starry flounder feed primarily on benthic fauna, including bivalves, polychaetes, brittle stars, and small crabs (Jewett & Feder, 1980; Miller, 1967; Orcutt, 1950). English sole also feed primarily on benthic fauna, including polychaetes, amphipods, and bivalves (Barry et al., 1996; Kravitz & Pearcy, 1976; Toole, 1980). The trophic position estimates in this study suggest that English sole were mainly feeding on primary consumers, such as bivalves, while starry flounder and shiner surfperch had similar trophic positions and were likely also feeding on secondary consumers to some extent. Rockfish with full stomachs had ingested a mixture of *Petrolithes*, *Cancer*, and *Majoid* crabs, *Heptacarpus* and *Pandalus* shrimp, and various fishes, including sculpins. This matches well with their estimated trophic position of >3 for all individuals and suggests that they were feeding on a mix of primary and secondary consumers. The fish δ_13_C values were similar to those of the bivalves, indicating primary reliance on pelagic carbon sources.

### Microplastic particle concentrations by species

The model estimates of MP digestive tract concentrations by species are within the range of those reported by other studies. The most extensive data exist for mussels, with one recent study finding averages of 0.20–0.94 particles g^‐1^ ww (or 1.25–15.42 particles ind^−1^) in wild *Mytilus* spp. collected from Italy, Spain, the Netherlands, Germany, France, Croatia, Denmark, and Tunisia for MPs down to 3 μm (Vinay Kumar et al., 2021). Our MP concentrations for mussels were higher in terms of ww, with corrected values of 0.70–28.14 particles g^‐1^ ww, but lower in terms of an individual, at 0.34–1.00 particles ind^−1^, which could be due to our inability to analyze smaller particles. It should be noted that the mussels sampled in our study were relatively small (0.9–2.9 cm in length), such that even a single MP in a mussel would result in a remarkably high ww concentration due to low body mass. Covernton et al. (2019) found mean concentrations of 0.10 particles ind^−1^ and 0.16 particles g^‐1^ dw for Manila clams for the same size range of particles, as compared to values of 0.29–0.71 particles ind^−1^ and 1.84–60.05 particles g dw^−1^ in the present study.

Mohsen et al. (2019) found that farmed sea cucumbers (*Apostichopus japonicus*) collected in China contained 0–30 MPs ind^−1^ in their digestive tracts compared with lower ranges of 0.39–0.99 and 0.28–0.88 MPs ind^−1^ observed here for Orange and California sea cucumbers, respectively. Xu et al. (2020) found that MP concentrations in crabs collected from Hong Kong beaches were significantly lower (mean 0.21 particles g^−1^ ww) in a predatory species (*Metopograpsus frontalis*) compared with two deposit‐feeding species (mean 2.84, 2.59 particles g^−1^ ww, *Austruca lacteal*, and *Macrophthalmus convexus*), although there were other species of both feeding types with intermediate concentrations. The crab species in our study were all predatory, which could explain the lower concentrations in comparison with the more contaminated Hong Kong crabs – 0.35 to 2.22, 0.45–0.57, and 0.09–0.68 particles g^−1^ ww for graceful rock, red rock, and Dungeness crabs, respectively, in addition to differences in environmental exposure. The sea cucumber and crab studies both used similar visual preselection methods, as was used in our study, so the size range of identified particles should be comparable among studies.

Rochman et al. (2015) reported a mean of 1.00 MP ind^−1^ in the digestive tracts of flatfish (*Citharichthys sordidus*), 0.30 particles ind^−1^ in yellowtail rockfish (*Sebastes flavidus*), and 0.00 particles ind^−1^ in vermillion rockfish (*Sebastes miniatus*) and copper rockfish (*S. caurinus*). Our concentration estimates for flatfish were 1.24–1.31 particles ind^−1^ (Starry flounder) and 0.92–1.00 particles ind^−1^ (English sole), and for rockfish they were 0.49–0.83 particles ind^−1^ (black rockfish) and 0.29–0.75 particles ind^−1^ (copper rockfish). However, because Rochman et al. (2015) did not consider particles <500 μm in size, our lower size limit of detection might account for our higher concentrations.

### Bioaccumulation

Bioaccumulation factors for MPs in digestive tracts decreased with trophic position, suggesting several orders of magnitude higher bioaccumulation for lower‐trophic‐level than higher‐trophic‐level animals. However, bioaccumulation is difficult to interpret in the context of transitory MPs in animal digestive tracts compared with its common usage for lipophilic chemical contaminants (Arnot & Gobas, 2006). For example, Dawson et al. (2018) exposed Antarctic krill (*Eupausia superba*) to polyethylene MP spheres in their food for 10 days followed by a 15‐day depuration and found that MP concentrations remained constant in the krill during the exposure, then decreased rapidly during depuration. They fed the krill for 4 h day^−1^ on MP‐contaminated food, followed by 20 h with no food, and sampled krill at Days 1, 2, 4, 7, and 10, after allowing sampled individuals to feed on uncontaminated algae for an additional 4 h. MP concentrations in the krill did not increase throughout their experiment, so the authors concluded that bioaccumulation was not occurring. However, the krill were unable to completely remove MPs before their next feeding period. Because bioaccumulation is established on the idea that ingestion rates are higher than excretion rates, this would suggest a low level of bioaccumulation rather than none. We thus propose that researchers should consider the consistent presence of MPs over time, in proportion to body size, as bioaccumulation of MPs in reference to digestive tracts, rather than an increase in concentration over time. The residence time of MPs in digestive tracts (and other tissues) determines the bioavailability of sorbed contaminants and residual monomers, and the exposure to these chemicals over time relative to body mass determines toxicity (Watanabe et al., 1992). Thus, this measure of bioaccumulation is applicable when considering the potential toxicity of MPs to an animal.

Our data imply at least an order of magnitude more bioaccumulation of MPs for suspension‐feeding bivalves and sea cucumbers compared to predatory species. Although we analyzed the bivalves' whole bodies, the individuals were small (0.9–2.9 cm length, 0.1–1.8 g ww), and it is unlikely that the size range of particles analyzed would have been present in other organs. The bioaccumulation factors of suspension and deposit‐feeding sea cucumbers were lower than the bivalves, but similar between the two sea cucumber species, despite different feeding habits. The bioaccumulation factors for predators, including crabs, sea stars, and fish, were at least an order of magnitude lower than bivalves and sea cucumbers for the crabs and rockfish.

Shiner surfperch had the highest bioaccumulation factors of the predator species, nearly as high as those for clams, followed by flatfish and sea stars, which were at the same order of magnitude as the sea cucumbers. Previous studies showed that planktivorous fish may selectively ingest MPs due to their similarity in size, shape, and sometimes color to typical prey items (Ory et al., 2017, 2018). This could explain why the flatfish and shiner surfperch in this study had high bioaccumulation factors—because these species all feed (to varying extent) on small animals of similar sizes to the observed MPs (e.g., zooplankton, benthic worms). The isotopic enrichment values of the flatfish and surfperch further support this, suggesting mainly pelagic carbon sources and feeding on secondary consumers (such as zooplankton). Shiner surfperch, which had especially high bioaccumulation factors, are a fast growing viviparous fish species that requires high weight‐specific food intake and empties its digestive tract every ~8 h (i.e., as compared to ~24 h for rockfish) (Gordon, 1963; Singer, 1985). Frequent consumption of prey that resemble MPs could therefore explain why shiner surfperch had higher bioaccumulation factors than the other fish species in this study. Nonetheless, these higher bioaccumulation factors do not necessarily suggest that MPs are persistent in shiner surfperch digestive tracts for extended periods of time, but rather that they are entering their bodies faster than (or at similar rates to) excretions rates and to a greater extent relative to their body mass than for the larger fish.

### Trophic transfer and biomagnification

Our results revealed that trophic transfer from prey to predator occurred within the two rockfish species, with evidence of (i) MPs in the bodies of ingested prey and (ii) higher MP concentrations in the digestive tracts of rockfish with full stomachs compared to those with empty stomachs. Copper rockfish have been shown to ingest 0.5%–3.7% of their body mass in food daily (Murie, 1995). Assuming these maximum/minimum food consumption percentages apply to copper and black rockfish and using the mean posterior for MP concentration in ingested animals by ww, we estimate a minimum intake of 0.03–0.10 particles day^−1^ and a maximum intake of 0.21–0.73 particles day^−1^ for the size range of rockfish that were sampled. These ranges suggest that, on average, an individual rockfish ingests less than one >100‐μm MP every day via trophic transfer. Rockfish require ~9–12 h to digest half of the food in a full stomach (Singer, 1985), meaning that all food should be processed through the digestive tract within ~24 h—allowing plenty of time for MPs to be excreted before the next meal. This could explain why MP concentrations for rockfish with empty stomachs were so low in this study. Thus, accumulation of MPs appears to be minimal in rockfish, further explaining why the models did not support the occurrence of biomagnification.

The digestive tracts of individual animals from all species had varying amounts of ingested food and debris, which is why we modeled digestive tract concentrations as particles per individual rather than particles per gram. When viewed in terms of either digestive tract or body weight, trophic dilution was occurring, since larger animals had numbers of MPs similar to those of smaller animals. The similar slopes for the relationship between MP concentration and trophic position, at each of the three sites, suggest that excretion occurs fast enough to prevent accumulation of MPs in digestive tracts. This lack of biomagnification in digestive tracts agrees with several recent reviews (Gouin, 2020; Miller et al., 2020; Walkinshaw et al., 2020). Other studies supplied evidence for the rapid excretion of MPs from digestive tracts, thereby limiting any biomagnification. Several studies using field‐collected samples also found no relationship between trophic level and MP concentrations in digestive tracts, gills, or muscle of various fish and invertebrate species (Akhbarizadeh et al., 2019; Bour et al., 2018; Filgueiras et al., 2020; Guven et al., 2018; Welden et al., 2018). Welden et al. (2018) found that plaice (*Pleuronectes platessa*) collected from the Celtic Sea had ingested sand eels (*Ammodytes tobianus*) containing MPs. However, there was no significant difference between prey and predator, suggesting a lack of MP retention. In contrast, a study by Zhang, Wang, et al. (2019) found a significant, positive correlation between MP concentrations in gastrointestinal tracts and gills and trophic level across 11 fish and 8 crustacean species collected from the East China Sea. However, the difference was <3 MPs between their highest and lowest sampled trophic levels.

Two studies also employed stable‐isotope analysis to measure MPs across trophic levels within food webs. Garcia et al. (2021) investigated a food web within the Garonne River in the southwest of France. They found that although digestive tract concentrations of 700‐ to 5000‐μm MPs did not increase with trophic position for freshwater fish, they did increase for macroinvertebrates. However, they concluded that biomagnification was unlikely because ingestion of MPs had no relationship with resource origin or feeding mode. Piarulli et al. (2020) examined benthic invertebrates from salt marsh sediments in three coastal lagoons on the northern Adriatic coast. They found that only 4% of individuals contained MPs, and the authors were unable to establish a relationship with trophic position. However, it seems likely that such a low degree of contamination would preclude any biomagnification. Several laboratory studies also demonstrated that, even under high MP exposure levels, excretion from the digestive tract is rapid and trophic dilution is the predominant outcome (Elizalde‐Velázquez et al., 2020; Kim et al., 2018; Sun et al., 2017). Alava (2020) used a modeling approach to provide evidence that biomagnification of MPs in a northeast Pacific food web, including large cetaceans, is unlikely to be occurring, further supporting our findings.

In this study, we also demonstrated that magnification of >100‐μm MPs in fish livers did not occur, with a mean trophic magnification factor of 0.52 instead suggesting trophic dilution. Akhbarizadeh et al. (2019) reported similar findings, calculating a trophic magnification factor of 0.72 in the gills and muscles of prawns (*Penaeus semisulcatus*), crabs (*Portunus armatus*), and fish (*Epinephelus coioides*, *Platycephalus indicus*, and *Liza klunzingeri*). It is likely that our 100‐μm lower size limit was not sufficient to quantify MPs in fish livers accurately and that only smaller particles would be able to translocate to this organ. Thus, our findings of any larger MPs in fish livers should be interpreted with some caution because the mechanism by which >100‐μm MPs could translocate to fish livers is unclear (Kim et al., 2020).

### Methodological considerations

We employed several new methods in this work that will be useful for studying MPs in food webs and other contexts. We demonstrated that stable‐isotope analysis was extremely useful for studying the movement of MPs within food webs. Applying this method more broadly will enable researchers to better quantify the dynamics of MPs within specific food webs. This study also represents the first application of a random forest model to classify unknown particles as synthetic or nonsynthetic, using expert user input and particle characteristics and, after training the model, using spectroscopically verified particles. For studies still relying on visual and physical methods to sort potential MPs, it is often difficult to transfer and analyze 100% of the particles. Simply excluding these particles is problematic, so researchers generally apply the proportion of analyzed particles that were MPs to correct the reported concentrations across all samples. However, this fails to consider subtleties relating to particle size, shape, color, or type of sample, and in addition ignores the ability of an experienced user to judge (or guess, to some degree) whether a particle might be plastic. Machine learning classification models offer the potential to classify these unknown particles, while considering user input to allow a more detailed analysis. On our fitted data, the random forest model correctly classified 75.1% of synthetic particles as synthetic and misclassified 24.9% as natural. The model classified 124 of 345 unknown particles (35.9%) as synthetic, compared to the comparable 32.5% of known particles classified as synthetic via spectroscopy, demonstrating the good performance of the model. While the overall numbers do not look different compared with a proportions approach, the random forest method allowed for these particles to be more specifically identified within sample types according to physical characteristics and user expertise.

Our use of Bayesian hierarchical models to incorporate uncertainty across levels of the analysis represents an approach that may be of broader use in the study of microplastics. To date, most researchers have accounted for background contamination of their samples using either simple deterministic blank subtraction (i.e., whereby the mean MP content in blanks is subtracted from sample counts) or by ignoring counts that are above the limit of either detection or qualification (Brander et al., 2020). The limit of detection is the mean MP count (or mass) from blanks (minimum of three replicates) plus two standard deviations. The limit of quantification is the limit of detection plus three standard deviations from the mean of blanks (maximum 10 replicates). However, because MP concentrations in samples are often low, we argue that limits of detection and quantification limit researchers' ability to make causal inferences by reducing the effective MP counts of many samples to zero. The Bayesian approach applied here assumes that background contamination will follow a Poisson distribution and allows for stochastic variation in blank subtraction (according to the type of sample) and, therefore, estimation of a distribution of “true” sample values. This approach will be of use in future MP research, which should compare the ability to make inferences around true MP sample numbers depending on the approach used to account for background contamination.

Like earlier work, the main limitation of this study is the inability to reliably analyze particles <100 μm in size. Thus, our conclusions can only be applied to the larger MP size fractions. Smaller particles may accumulate at higher concentrations, both in the digestive tracts and tissues of fish, and even possibly biomagnifying, though it is unlikely that the larger, >100‐μm particles measured by our study translocate out of digestive tracts (Carr et al., 2012; Roch et al., 2020). Though it seems likely that small micro‐ and nanoplastics will be excreted from digestive tracts along with larger MPs, the risk of translocation—and therefore accumulation—in other tissues is higher. Emerging technologies are beginning to allow for the analysis of smaller MPs. Automated Raman and FTIR spectroscopy have the potential to detect and classify particles down to 1–5 or 10–20 μm, respectively (Primpke et al., 2020). Thermal degradation methods followed by gas chromatography‐mass spectrometry analysis of pyrolysis products can quantify even smaller particles, although this is currently limited to concentration by weight according to polymer, with particle counts not yet possible. Further research combining methodologies like those employed here with some of these newer techniques will be essential for determining whether smaller micro‐ and nanoplastics and associated chemicals biomagnify in food webs.

## CONCLUSIONS

Our results suggest that smaller, lower‐trophic‐level animals may be at the greatest risk of any potential health effects caused by MP ingestion given higher exposure rates relative to their body size, especially at urbanized locations where exposure may be higher. Although we cannot completely rule out biomagnification of MPs under high‐exposure scenarios or for MPs <100 μm, our findings from rockfish stomachs indicate that excretion appears to be fast enough to limit accumulation and to make magnification in digestive tracts unlikely. Preliminary risk assessments have proposed that under current MP exposure scenarios, few organisms are likely to experience any significant health effects in aquatic environments (Everaert et al., 2020; Koelmans et al., 2020). However, these assessments rely on acute exposure toxicity experiments and do not consider ingestion rates or the effects of chronic exposure. Future ecological risk assessments will need to consider these factors, including modeling the food‐web dynamics of MPs. Our work provides a significant contribution to efforts to explore the ecological risk of MPs by modeling the relationship between trophic position and digestive‐tract MP concentrations among diverse animals in coastal marine food webs, and by directly quantifying trophic transfer of MPs to rockfish—relatively high‐trophic‐level long‐lived species. Our use of Bayesian methods for exploring relationships between MP concentrations and ecological traits, while probabilistically accounting for background contamination by expanding uncertainty intervals, provides an additional framework for realistically exploring ecological risk.

## CONFLICT OF INTEREST

The authors declare no competing interests.

## Supporting information


Appendix S1

## Data Availability

Data and code (GCov, 2022) are available in Zenodo at https://doi.org/10.5281/zenodo.5829446.
