# Supplementary material for: Large size (>100‐μm) microplastics are not biomagnifying in coastal marine food webs of British Columbia, Canada
Source: Ecol Appl. 2022 Jul 4;32(7):e2654. doi: 10.1002/eap.2654 (PMC9786919; doi:10.1002/eap.2654)
Supplement: Supplementary file 1 — Appendix S1 [file EAP-32-e2654-s001.pdf]

Large size (>100-μm) microplastics are not biomagnifying in coastal marine food webs of British Columbia, Canada

Garth A. Covernton, Kieran D. Cox, Wendy L. Fleming, Brittany M. Buirs, Hailey L. Davies, Francis Juanes, Sarah E. Dudas, John F. Dower

Ecological Applications

Appendix S1

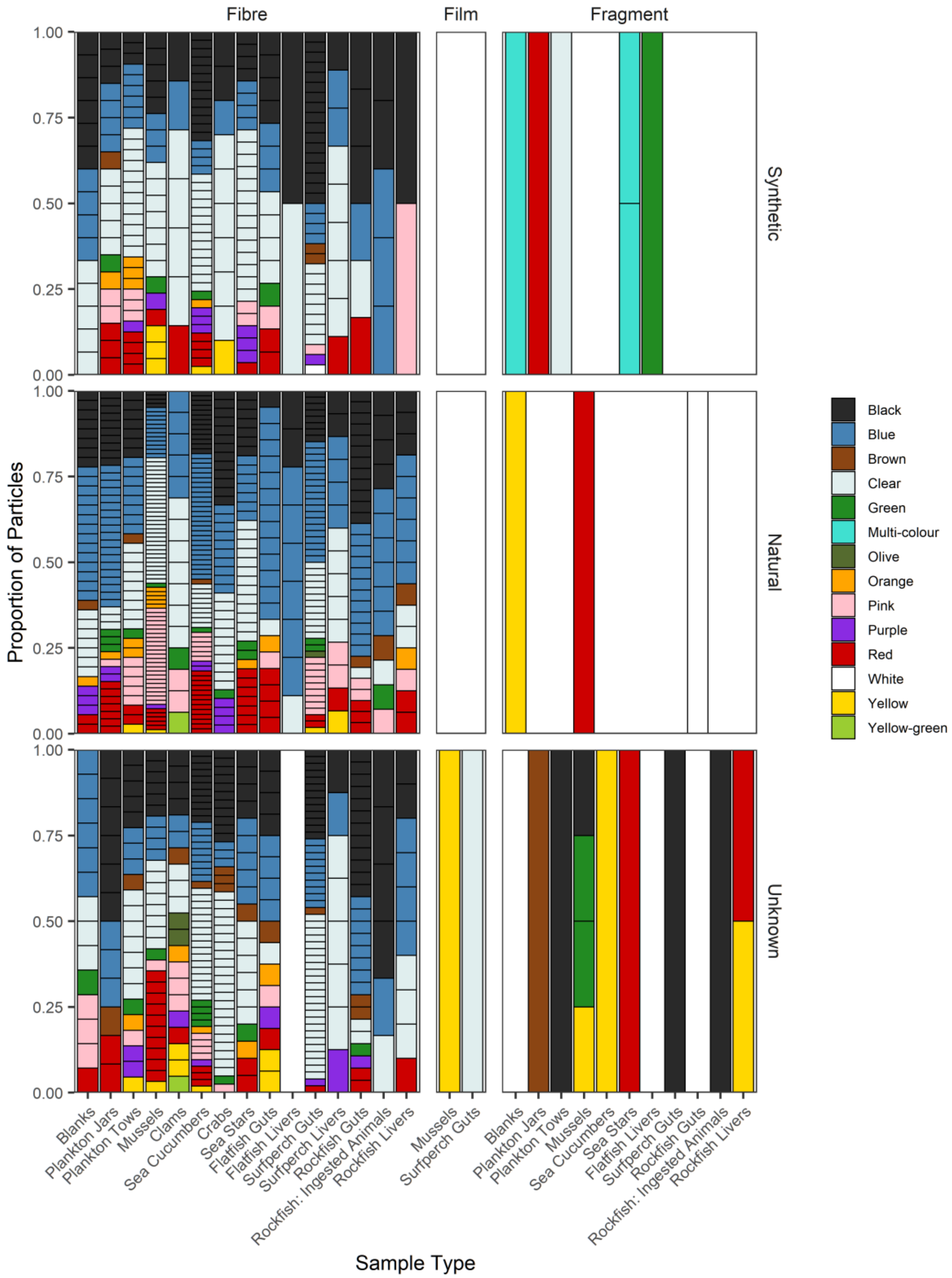

Figure S1: Colours of all potential microplastic particles, by sample type (including blanks), particle class, and particle shape. Each box within the bars indicates a single particle.

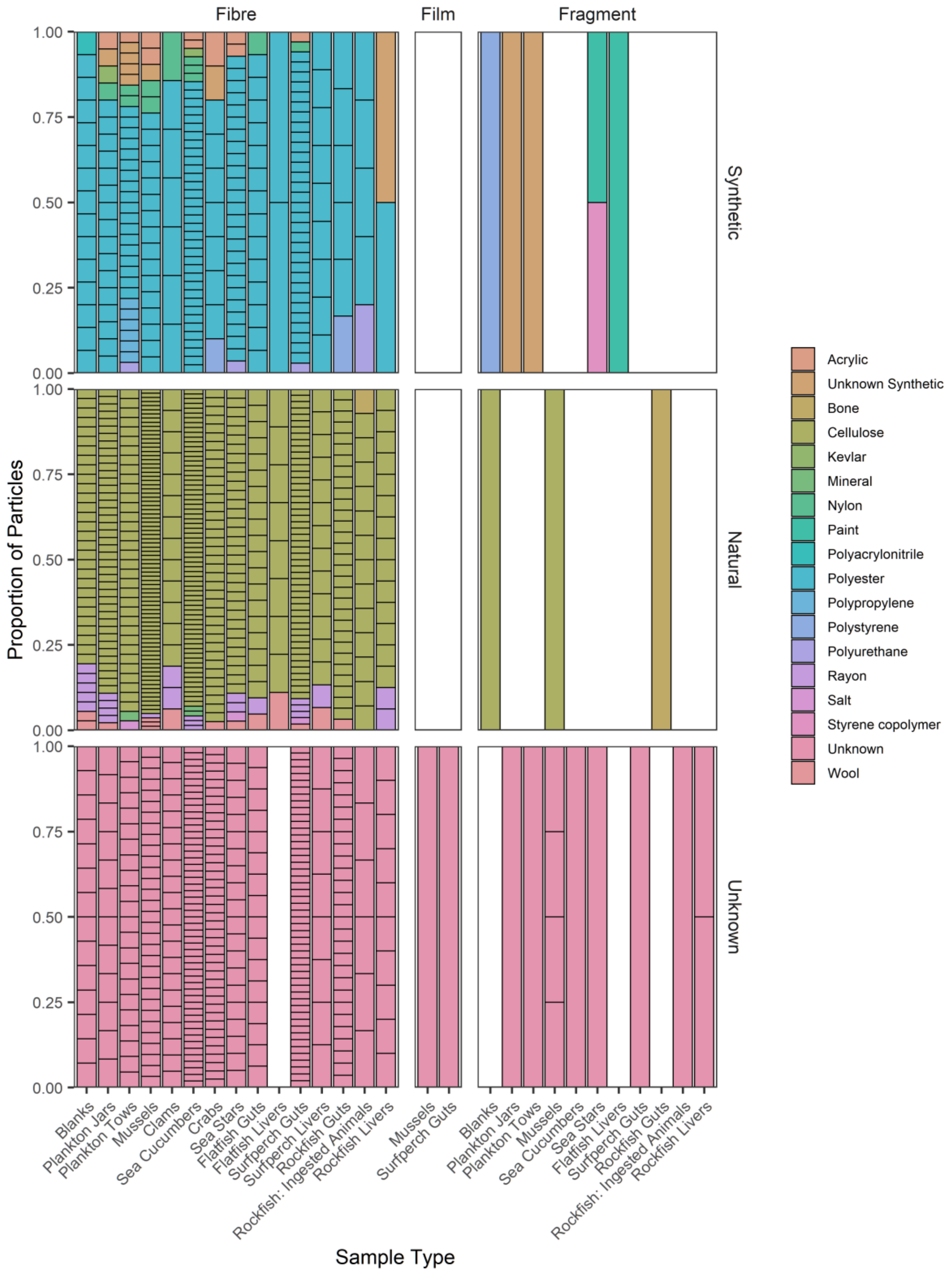

Figure S2: Particle classifications for the 882 particles for which Raman spectroscopy was attempted. Of these particles, 779 were successfully classified as either synthetic polymers (microplastics), natural (salt, bone, mineral, or clear cellulose), natural anthropogenic (dyed cellulose or wool), or semi-synthetic (rayon) particles. Each box within the bars indicates a single particle.

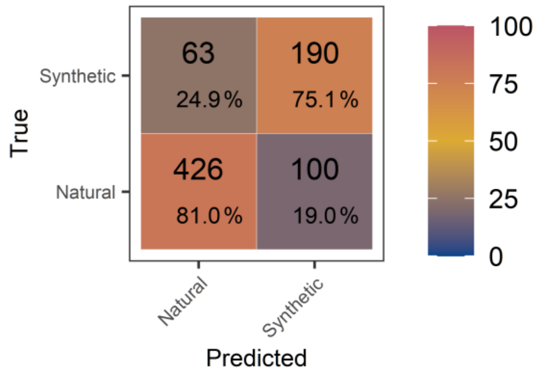

Figure S3: Confusion matrix from the random forest model. Each box contains the number of particles that were correctly or incorrectly classified into each category and the percent of each particle type that were classified into each group. The boxes are coloured according to these percentage values.

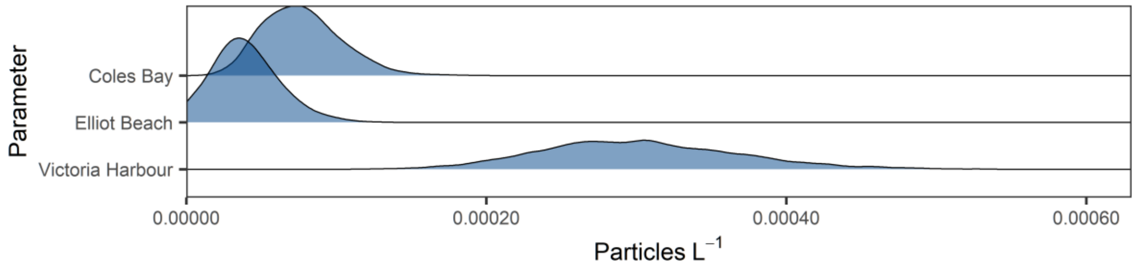

Figure S4: Parameter posteriors for the plankton tow GLMM with microplastic count in each sample as the response variable, site as a predictor, and sample volume as an offset term. Parameter estimates are exponential transformed to be on the scale of the response variable.

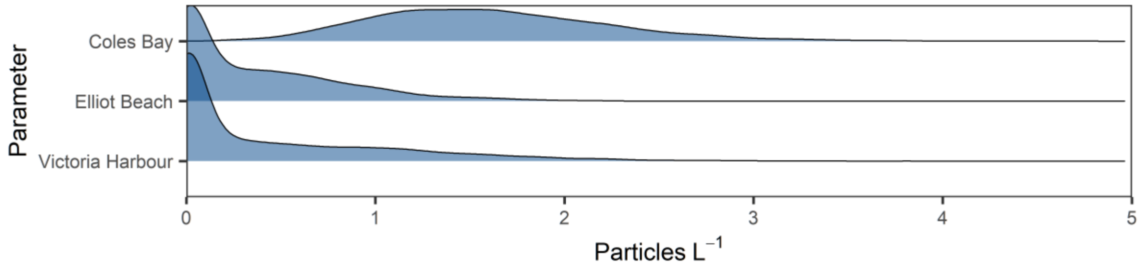

Figure S5: Parameter posteriors for the seawater jar sample GLMM with microplastic count in each sample as the response variable and site as a fixed effect. Parameter estimates are exponential transformed to be on the scale of the response variable.

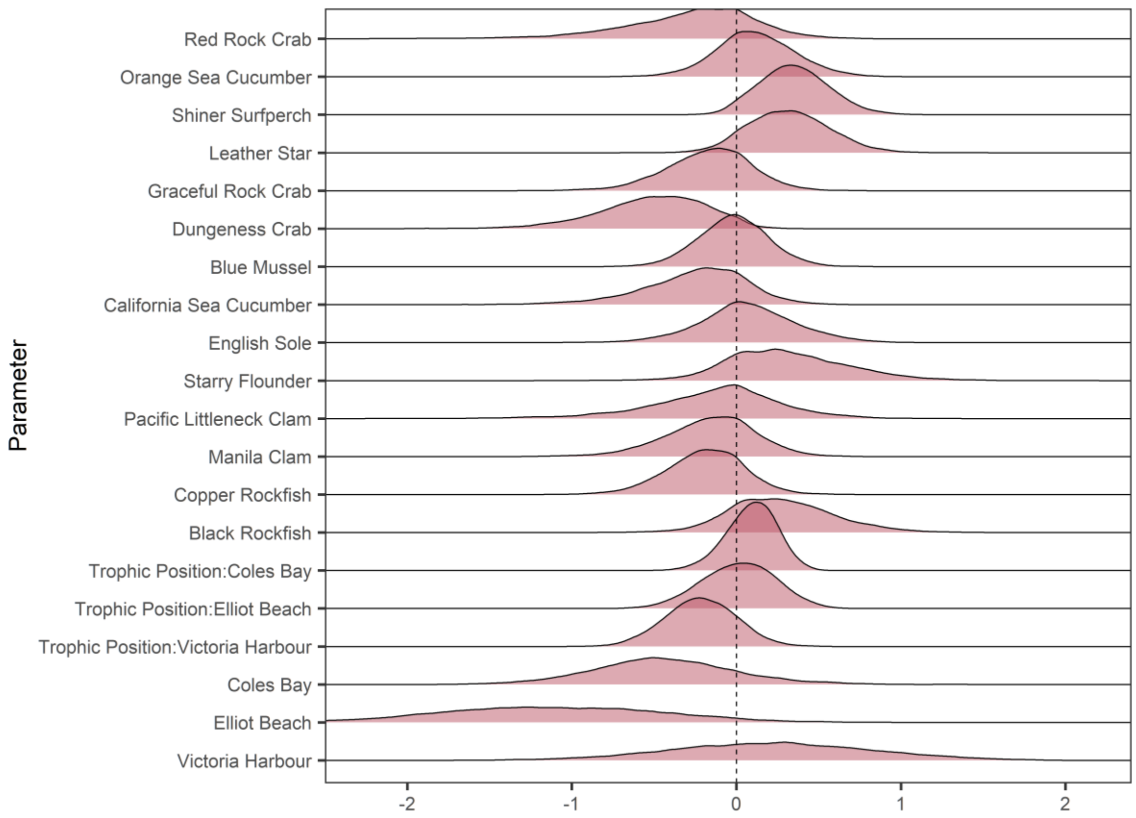

Figure S6: Parameter posteriors for the animal digestive tracts GLMM with number of microplastics in each digestive tract as the response variable, site and the interaction between site and trophic position as fixed effects, and species as a random effect. Parameter estimates on are on the scale of the link function (log-scale).

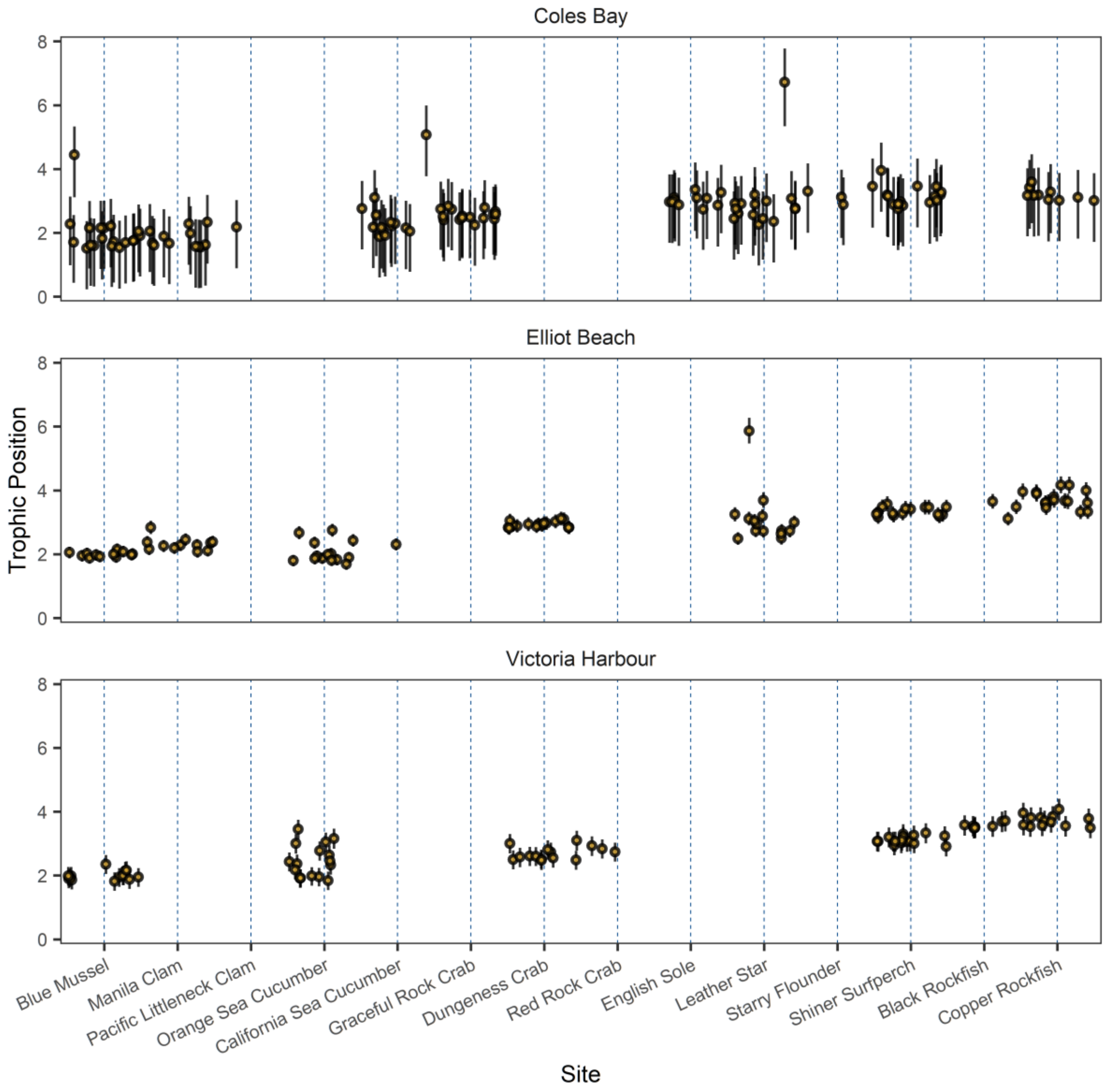

Figure S7: Predicted trophic position for each sampled individual according to the digestive tracts and trophic position GLMM. The points represent the mean of the posterior predictive samples and the lines the 95% credibility interval for each individual of each species.

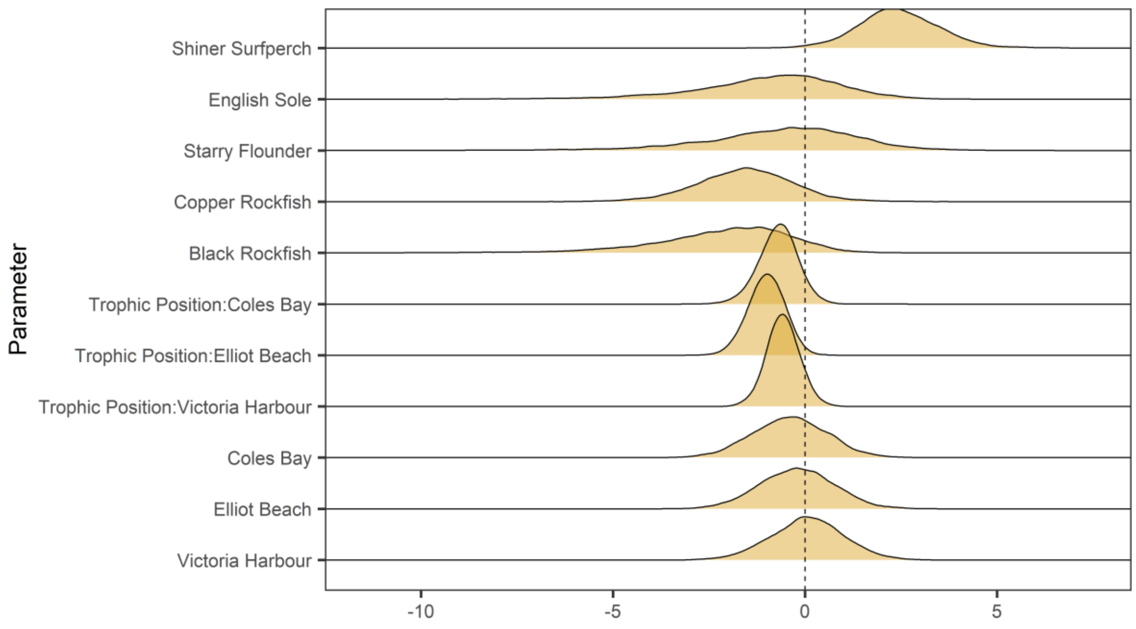

Figure S8: Posteriors for the fish liver GLMM with number of microplastics in a liver as the response variable, site and the interaction between site and trophic position as fixed effects, and species as a random effect. Parameter estimates on are on the scale of the link function (log-scale).

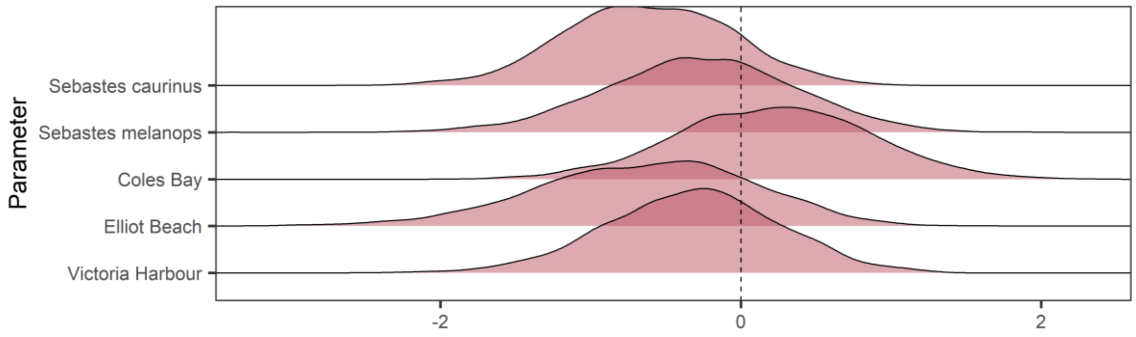

Figure S9: Posteriors for the GLMM quantifying microplastics in animals ingested by the rockfish with number of microplastics in a sample as the response variable and site and species as fixed effects. Parameter estimates on are on the scale of the link function (log-scale).

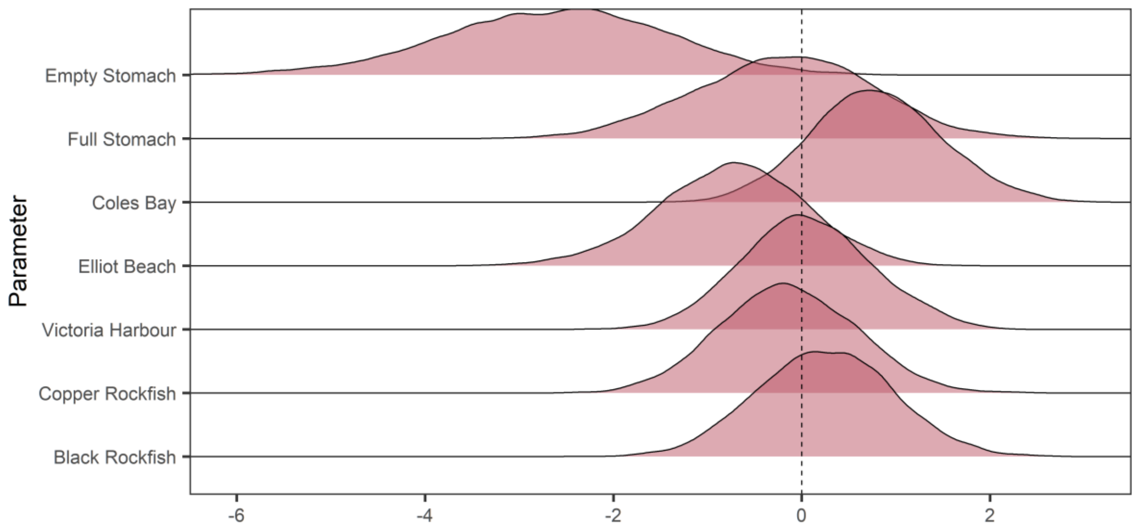

Figure S10: Posteriors for the rockfish digestive tracts GLMM with number of microplastics in a digestive tract as the response variable and fish total length, trophic position, stomach fullness (empty or full), site, and species as fixed effects. Parameter estimates on are on the scale of the link function (log-scale).
